# Prevalence and prognostic value of preexisting sarcopenia in patients with mechanical ventilation: a systematic review and meta-analysis

**DOI:** 10.1186/s13054-022-04015-y

**Published:** 2022-05-16

**Authors:** Tingting Jiang, Taiping Lin, Xiaoyu Shu, Quhong Song, Miao Dai, Yanli Zhao, Li Huang, Xiangping Tu, Jirong Yue

**Affiliations:** Department of Geriatrics and National Clinical Research Center for Geriatrics, West China Hospital, Sichuan University, Chengdu, 610041 Sichuan Province China

**Keywords:** Mechanical ventilation, Prevalence, Prognosis, Sarcopenia, Systematic review

## Abstract

**Background:**

Sarcopenia is defined as age-related loss of muscle mass, strength, and/or function in the context of aging. Mechanical ventilation (MV) is one of the most frequently used critical care technologies in critically ill patients. The prevalence of preexisting sarcopenia and the clinical impact of its prognostic value on patients with MV are unclear. This review sought to identify the prevalence and prognostic value of preexisting sarcopenia on MV patient health outcomes.

**Methods:**

Relevant studies were identified by searching MEDLINE, Embase, and the Cochrane library and were searched for all articles published as of December 2021. The prevalence of sarcopenia was determined using the authors' definitions from the original studies. Comparisons were made between patients who did and did not have sarcopenia for prognostic outcomes, including mortality, the number of days of MV, the length of intensive care unit stay, and the length of hospital stay. Odds ratios (ORs) and weighted mean differences with 95% confidence intervals (CIs) were used for pooled analyses of the relationships between sarcopenia and prognostic outcomes.

**Results:**

The initial search identified 1333 studies, 17 of which met the eligibility criteria for the quantitative analysis, including 3582 patients. The pooled prevalence was 43.0% (95% CI 34.0–51.0%; *I*^2^ = 96.7%). The pooled analyses showed that sarcopenia was related to increased mortality (OR 2.13; 95% CI 1.70, 2.67; *I*^2^ = 45.0%), longer duration of MV (MD = 1.22; 95% CI 0.39, 2.05; *I*^2^ = 97.0%), longer days of ICU stay (MD = 1.31; 95% CI 0.43, 2.19; *I*^2^ = 97.0%), and hospital stay (MD 2.73; 95% CI 0.58, 4.88; *I*^2^ = 98.0%) in patients with MV.

**Conclusion:**

The prevalence of sarcopenia is relatively high in patients with MV, and it will have a negative impact on the prognosis of patients. However, further, large-scale, high-quality prospective cohort studies are required.

**Supplementary Information:**

The online version contains supplementary material available at 10.1186/s13054-022-04015-y.

## Introduction

Sarcopenia, a syndrome characterized by low muscle mass (LMM) and low muscle strength (LMS) and/or by low physical function (LPF) [1, 2], is a key cause of frailty among older adults and contributes to increasing in fall rates, fractures, poor quality of life, and mortality [3, 4]. An estimated 5–13% of 'healthy' older individuals [1, 5] and 30–70% of intensive care unit (ICU) patients are thought to be affected by this condition [6, 7].

Sarcopenia onset may be linked to age, nutrition, activity levels, and the incidence of certain conditions, including chronic inflammatory diseases and cancer [8]. Sarcopenia additionally has a number of adverse social and economic impacts, accounting for 1.5% of overall healthcare expenditures in recent years [9]. Approaches to treating sarcopenia can consist of exercise, hormone therapy, and nutrition-focused interventions. Early identification and timely intervention of sarcopenia can help to more effectively treat this co-alleviating condition and reduce complications.

Mechanical ventilation (MV) is one of the most commonly used life support techniques in clinical practice [10]. Approximately 30–88% of critically ill patients require MV [11]. It is now widely recognized that MV may adversely affect muscles [12]. Following MV, critically ill patients exhibit both acute and persistent decreases in muscle mass, termed ICU-acquired weakness, tied to reductions in physical function and overall quality of life [13–16]. However, due to the chronic inflammatory reaction and lack of exercise, the prevalence of preexisting sarcopenia, rather than ICU-acquired weakness, is also very high in patients with MV. Some studies have found that such patients on MV with preexisting sarcopenia tend to have poor outcomes, including increased ICU mortality, prolonged duration of MV, ICU stay, and hospital stay [7, 17–19]. Other studies have reported inconsistent outcomes and conclusions [20, 21]. There have been systematic reviews [22, 23] about sarcopenia and mortality in critically ill patients, but these studies focused on critically ill patients. Compared with the vague definition of critically ill patients, patients with MV may be more clearly defined. In addition, respiratory myasthenia associated with sarcopenia may be more relevant to the outcome of mechanical ventilation.

Although systematic reviews and meta-analyses exploring the prevalence and prognosis of ICU-acquired weakness have been performed, no corresponding studies of the prevalence and impact of preexisting sarcopenia on prognosis in patients with MV have been published. This underscores the importance of conducting a definitive review to understand the prevalence of sarcopenia and its prognostic value in patients with MV. We thus performed a systematic review and meta-analysis of the prevalence and prognostic relevance of sarcopenia in surviving patients who underwent MV.

## Methods

### Search strategy

This systemic review was conducted in line with the Preferred Reporting Items for Systematic Reviews and Meta-Analyses 2020 statement (PRISMA 2020) principles [24] (Additional file 1: Table S1) and was registered on PROSPERO (registration number: CRD42021257376) in June 2021. We searched all the literature via Ovid from inception through the end of December 2021. The databases searched included MEDLINE, Embase, The Cochrane Database of Systematic Reviews, and the Cochrane Central Register of Controlled Trials. See Additional file 1: Table S2 for full search strategy details. The references of identified articles were additionally subjected to manual review to identify other relevant studies.

### Inclusion and exclusion criteria

The PICOS principle was used to establish study eligibility [24, 25]. The inclusion criteria were as follows: (1) patient: patients with MV, which were defined as adult patients (≥ 18 years old) who were admitted to the ICU department and underwent MV for ≥ 24 h; (2) exposure: sarcopenia, defined as the presence of LMM alone and/or LMS, LPF; sarcopenia diagnosed before MV or within 72 h after MV; (3) outcomes: reported the prevalence of sarcopenia or the clinical outcomes; and (4) study design: observational (cohort) and cross-sectional studies. Studies were excluded if (1) patients had ICU-acquired weakness; (2) case reports, reviews, or abstracts lacked complete data, as well as studies not published in English.

### Study selection and data extraction

Two reviewers (TTJ and XYS) independently screened the titles and abstracts to select relevant studies, with a full-text review being conducted when a given abstract was considered of potential relevance. Disagreements were resolved through discussion and consensus with a third investigator (JRY). Two investigators (TTJ and TPL) then extracted relevant data independent of one another using a standardized form, with the resultant data then being checked by the third reviewer (JRY). Data extracted from each study included author, year of publication, country, sample size, study design, patient demographics, diagnostic criteria of sarcopenia, follow-up duration, background diseases, and outcomes in individual groups.

### Assessment of quality

The Newcastle–Ottawa Scale (NOS) was independently used by two investigators (TTJ and QHS) to assess the quality and methodological strength of the selected studies [26]. Possible scores ranged from 2 to 9 stars, with 0–4, 5–6, and 7–9 stars corresponding to studies of poor, moderate, and high quality, respectively.

### Outcome measures

The analyzed primary outcomes were as follows: (1) prevalence of sarcopenia in patients with MV. Sarcopenia prevalence is defined as exit sarcopenia before MV or within 72 h after MV, measured with validated sarcopenia diagnostic criteria; and (2) all-cause mortality after MV in patients with sarcopenia, which included ICU mortality, in-hospital mortality, 30-day mortality, and ≥ 3-month mortality (including 90-day and 120-day mortality).

Secondary outcomes included length of ICU stay (ICU LOS), duration of MV, and length of hospital stay (LOS). According to standard use, ICU LOS was defined as the total number of days the patient was in the ICU. Overall LOS was the number of days the patient was hospitalized (from inpatient admission to discharge). The duration of MV was the total time from intubation or tracheotomy to extubation.

### Statistical analysis

The main outcomes were determined using STATA/SE (version 14.0, StataCorp, TX, USA). Secondary outcomes were analyzed using Review Manager (Version 5.4, The Cochrane Collaboration, Oxford, UK). *P* < 0.05 was considered significant for all analyses. The *I*^2^ statistic was used to analyze heterogeneity, and pooled analyses of sarcopenia prevalence were performed with a random-effects model in the presence of significant heterogeneity (*I*^2^ ≥ 50%), with fixed-effects models otherwise being utilized. The effects of sarcopenia on prognostic outcomes (e.g., mortality) were assessed by retrieving the odds ratios (ORs) and the 95% confidence intervals (CIs) to conduct meta-analyses if possible. When these values were not available from multivariate analyses, data from univariate analyses were instead obtained to conduct meta-analyses (Additional file 1: Table S3). For continuous data (e.g., the duration of MV, the days of ICU LOS, and the hospital LOS.), the weighted mean differences (WMDs) with 95% CIs were used for outcomes pooled.

### Subgroup analysis and meta-regression

For primary outcomes, we conducted subgroup analyses on diagnostic criteria (including LMM alone or LMM plus LMS), different computed tomography (CT) scan sites, and background diseases (including surgical and internal diseases) for prevalence. In addition, we also performed subgroup analysis on mortality at different periods, including ICU mortality, in-hospital mortality, 30-day mortality, and ≥ 3-month mortality (including 90-day and 120-day); different diagnosis methods, including CT and BIA; and different types of diseases, including surgical diseases and internal diseases. For secondary outcomes such as duration of MV, ICU LOS, and LOS, we only performed subgroup analyses on different diagnosis methods [CT and BIA (bioelectrical impedance analysis) plus HGD (handgrip dynamometry)] because of the limited number of included studies. Meta-regression was conducted to assess whether the average age could affect sarcopenia prevalence and all-cause mortality.

### Sensitivity and publication bias analysis

Sensitivity analyses were used to establish the reliability and quality of the results by iteratively omitting single studies from pooled analyses. The Egger’s test [27] and Begg’s test [28] were used to assess publication bias (*P* < 0.05).

## Results

### Study selection

**Fig. 1:**
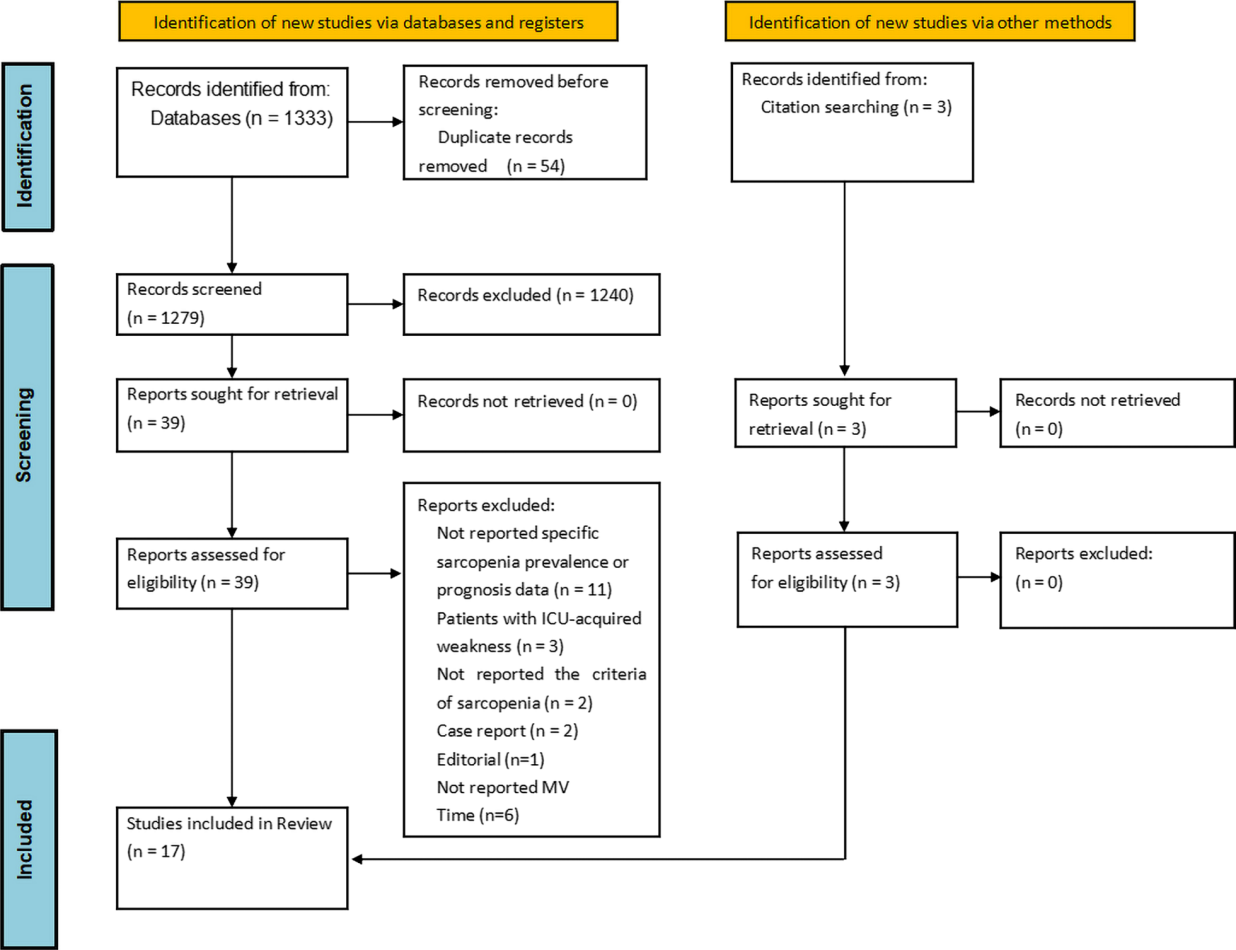
Flowchart of search results and study selection

Our initial search strategy identified 1333 studies of potential relevance, of which 54 were duplicates and 1240 were excluded following title and abstract review. Full-text review was performed for the remaining 39 studies, of which 25 were excluded in light of defined inclusion criteria (Additional file 1: Table S4). Three additional studies were identified through manual reference review. Finally, we selected 17 studies [7, 18–20, 29–41] that met our criteria for inclusion in the present systematic review and meta-analysis. The study selection flowchart is shown in Fig. 1.

### Study characteristics

The characteristics of the 17 included studies are summarized in Table 1. Of these, 3 adopted a prospective cohort study [30, 36, 38], 13 were retrospective cohort study [7, 19, 20, 29, 31–35, 37, 39–41], and 1 was a cross-sectional study [18]. The sample sizes for the included studies ranged from 45 to 519, with 3582 total patients and an average age ranging from 41.4 to 79 years. Among them, 12 and 5 studies were performed in surgical patients and patients with internal diseases, respectively. Patients were from diverse populations, with ten studies performed in Asia, five studies in America, and two studies in Europe (Table 1).

**Table 1:** Study and patient characteristics of included studies

References	Country	Design	Study interval	Sample size	Age/years (mean ± SD)	Male, *n* (%)	Background diseases	Prevalence (%)	Outcomes	Sarcopenia criteria	Diagnostic method
Doolittle et al. [20]	USA	Retrospective cohort study	2009–2017	238	60.4 ± 17.4	160 (67.2%)	Trauma	36.9	MV time, ICU LOS, LOS	LMM	CT
Moctezuma-Velázquez et al. [35]	Mexico	Retrospective study	2020.2–2020.5	519	51 ± 3.17	332 (64.0%)	COVID-19	22.0	Mortality, ICU admission	LMM	CT
Moon et al. [41]	South Korea	Retrospective study	2016.8–2018.12	190	78 ± 1.33	113 (59.5%)	Sepsis	50.5	ICU LOS, ICU and hospital mortality, LOS	LMM	CT
Han et al. [29]	Korea	Retrospective study	2015.1–2015.6	311	67.3 ± 14.9	180 (57.9%)	CAP	28.9	All-cause in-hospital mortality, LOS, vasopressor use, Ventilator weaning failure	LMM	CT
Vongchaiudomchoke et al. [30]	Thailand	Prospective, cohort study	2018.6–2019.12	120	75.1 ± 7.6	62 (51.7%)	Abdominal surgery	33.3	MV time, LOS, ADL, 120-day mortality	LMM + LMS	BIVA, HGD
Xi et al. [34]	China	Retrospective study	2010.1–2020.4	451	41.4 ± 15.9	373 (82.7%)	Trauma	24.8	MV time, ICU LOS, LOS, overall complications, 28-day and 90-day mortality	LMM	CT
Yuenyongchaiwat et al. [38]	Thailand	Prospective cohort study	2018	160	61.1 ± 11.5	90 (60.0%)	Heart surgery	26.9	MV time, LOS	LMM + LMS	BIA, HGD
Joyce et al. [37]	Australia	Retrospective observational study	2018–2019	279	63.7 ± 16.4	163 (58.4%)	Sepsis	68	30-day mortality, ICU-mortality, ICU LOS, LOS	LMM	CT
Ng et al. [33]	Malaysia	Retrospective observational study	2016.1–2016.12	228	54.4 ± 17.8	148 (64.9%)	Surgery	50	ICU mortality, In-hospital mortality, MV time, ICU sLOS, LOS	LMM	CT
Woo et al. [31]	Korea	Retrospective cohort study	2014–2019	45	66.4 ± 14.5	28 (62.2%)	Surgery	24.4	MV time, ICU LOS	LMM	CT
Kou et al. [40]	China	Retrospective cohort study	2013–2014	96	73.0 ± 2.97	63 (65.6%)	All kinds of surgery	31.30	The rate of DTW; ICU mortality	LMM	CT
Ji et al. [32]	China	Retrospective cohort study	2012.08.01–2016.07.31	236	68.75 ± 4.17	139 (58.9%)	Surgery	48.3	30-day mortality, ICU LOS, LOS, and hospital costs	LMM	CT
Akahoshi et al. [39]	Japan	Retrospective observational study	2012.05–2015.04	84	49.95 ± 16.3	47 (56.0%)	Trauma	29.7	30-day mortality, ICU LOS, LOS	LMM	CT
Ebbeling et al. [36]	USA	Prospective cohort study	2005–2010	180	74 ± 3.17	103 (57.0%)	Trauma	50.0	In-hospital mortality, MV time, ICU LOS, LOS	LMM	CT
Sheean et al. [18]	USA	Cross-sectional study	NR	56	58.5 ± 14.6	32 (57.1%)	Infection/Sepsis	58.60	NR	LMM	CT
Weijs et al. [19]	Netherlands	Retrospective cohort study	2003.12–2012-09	240	59.5 ± 17.8	201 (83.7%)	Trauma	63.0	ICU, 28-day and hospital mortality; MV time, ICU LOS, LOS	LMM	CT
Moisey et al. [7]	USA	Retrospective cohort study	2009–2010	149	79 ± 2.7	85 (57.0%)	Trauma	71.0	Ventilator-free days, ICU-free days, and ICU mortality	LMM	CT

*ADL* activities of daily living, *BIA* bioelectrical impedance analysis, *BIVA* bioelectrical impedance vector analysis, *CAP* community-acquired pneumonia, *COVID-19* coronavirus disease 2019, *CT* computed tomography, *DTW* difficult-to-wean, *HGD* handgrip dynamometry, *ICU* intensive care unit,* ICU LOS* the Length of intensive care unit stay, *LMS* low muscle strength, *LOS* length of hospital stay, *LMM* low muscle mass, *MV* mechanical ventilation, *NR* not reported

In terms of the definition of sarcopenia, it was diagnosed based solely on LMM in 15 studies. CT scans were utilized for muscle mass assessments. Twelve studies measured total skeletal muscle mass at the L1, L3, or L4 lumbar vertebrae [7, 18–20, 29, 31–34, 36, 37, 39]; two studies defined sarcopenia by measuring the thoracic paravertebral muscle (TPM) (T4 or T12) [35, 41]; one study defined sarcopenia based upon measurements of total psoas muscle area (TPA) [40]; and two studies defined sarcopenia based upon LMM (assessed by BIA) plus LMS (assessed by HGD) [30, 38]. A range of measurement approaches and cutoff thresholds were employed to detect the LMM and LMS and are presented in Additional file 1: Table S5.

### Risk of bias

The NOS was used to evaluate study quality and is shown in Additional file 1: Table S6. Overall, studies were of moderate quality, with a range of 2–8 scores. There were eight high-quality studies with scores of 7–8 [7, 19, 30–33, 37, 40].

### Sarcopenia prevalence

**Fig. 2:**
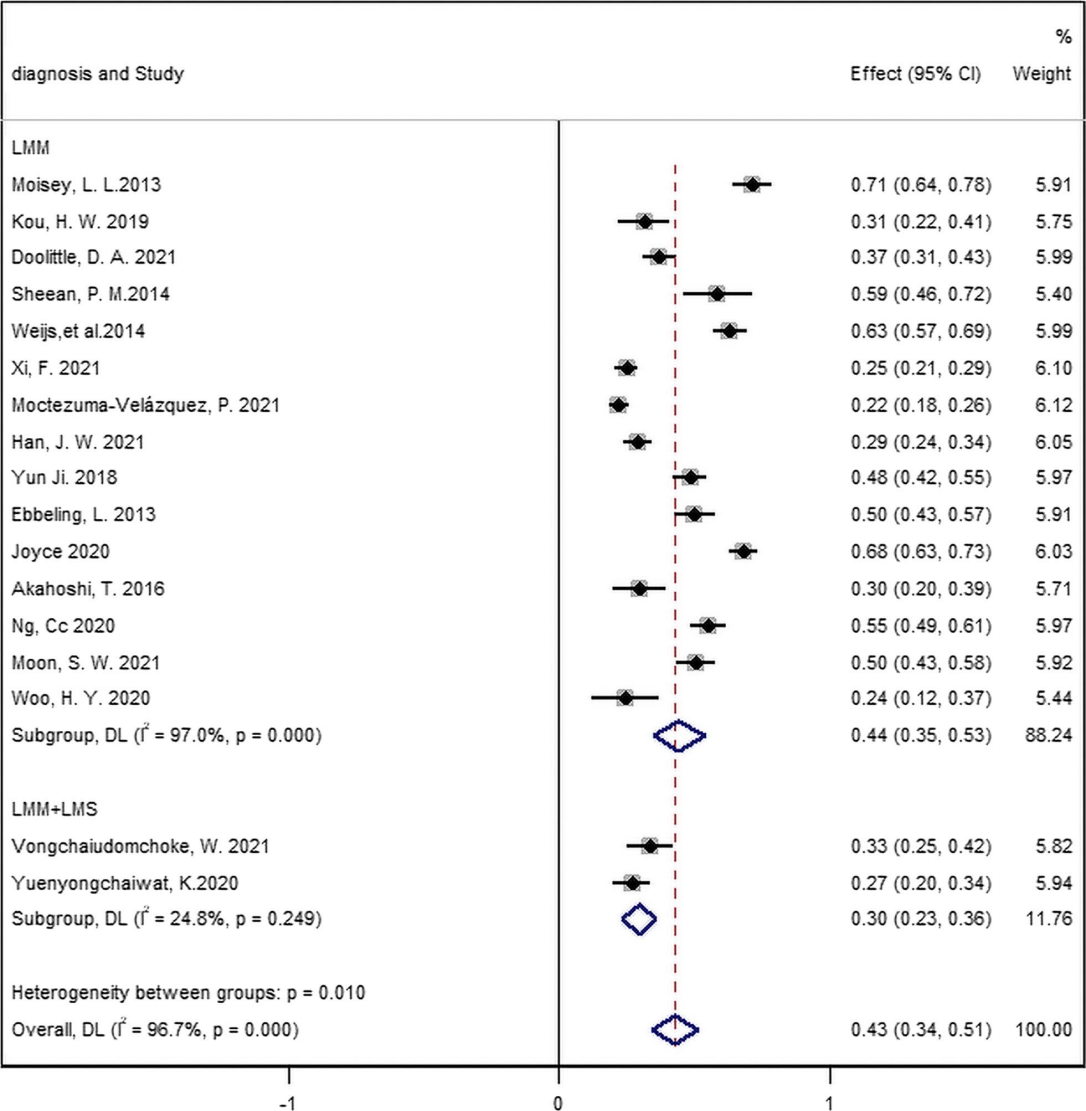
Prevalence of sarcopenia in patients with mechanical ventilation

Sarcopenia prevalence ranged widely among these 17 studies from 22 to 71.1% (Table 1), and the pooled estimate sarcopenia prevalence in patients with MV was 43.0% (95% CI 34.0–51.0%; *I*^2^ = 96.7%; Fig. 2).

### Subgroup analysis of prevalence

**Fig. 3:**
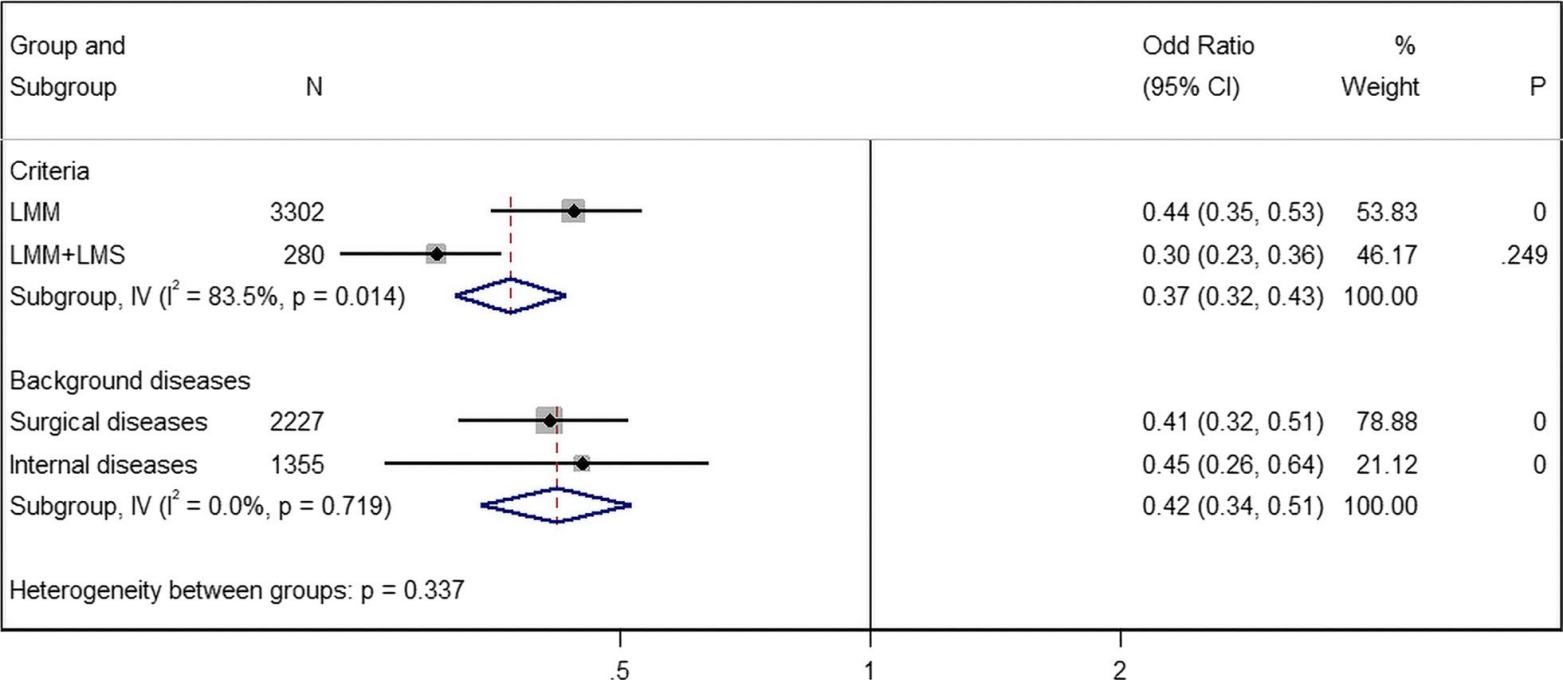
Subgroup analysis of sarcopenia prevalence by criteria and background diseases

Subgroup analyses revealed that the prevalence of sarcopenia in studies using only LMM assessed by CT scan [LMM; 44.0% (95% CI 35.0–53.0%, 15 studies, 3302 cases)] was higher than those in which it was defined based upon combination criteria assessed by BIA and HGD [LMM + LMS; 30.0% (95% CI 23.0–36.0%, 2 studies, 280 cases)] (Figs. 2, 3). For the different CT scan sites, the prevalence of sarcopenia diagnosed by paralumbar muscles (PLM) was 47.0% [95% CI 36.0–57.0%, 12 studies, 2497 cases], followed by TPM [36.0% (95% CI 8.0–64.0%, 2 studies, 709 cases)], and finally TPA [31.0% (95% CI 22.0–41.0%, 1 study, 96 cases)] (Additional file 1: Fig. S1). Additionally, sarcopenia prevalence was higher in internal diseases [45.0% (95% CI 26.0–64.0%, 5 studies, 1355 cases)] than in surgical diseases [41.0% (95% CI 32.0–51.0%, 12 studies, 2227 cases)], although the difference was not significant (*P* = 0.719, Fig. 3).

### Meta-regression of prevalence

Pooled average age data indicated that it had no impact on sarcopenia prevalence in a meta-regression analysis [regression coefficient 0.005 (95% CI − 0.003 to 0.013), *P* = 0.165, 17 studies, 3582 cases] (Additional file 1: Fig. S2).

### Effects of sarcopenia on mortality

**Fig. 4:**
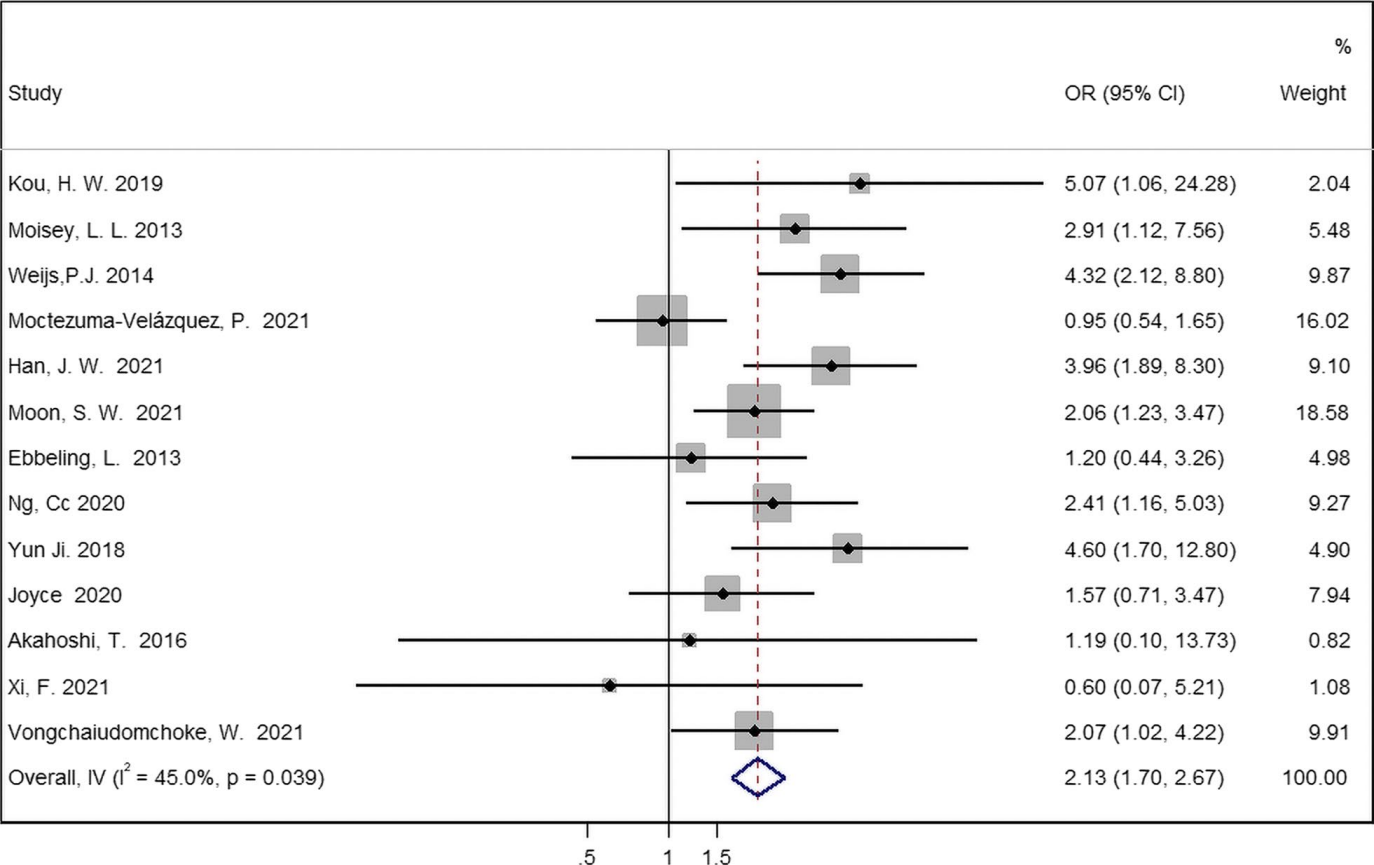
All-cause mortality of patients with mechanical ventilation

Data from 13 studies, including 3079 participants, were available to meta-analyze all-cause mortality. Preexisting sarcopenia was related to a higher risk of mortality (OR 2.13; 95% CI 1.70, 2.67; *I*^2^ = 45.0%; Fig. 4).

### Subgroup analysis of mortality

**Fig. 5:**
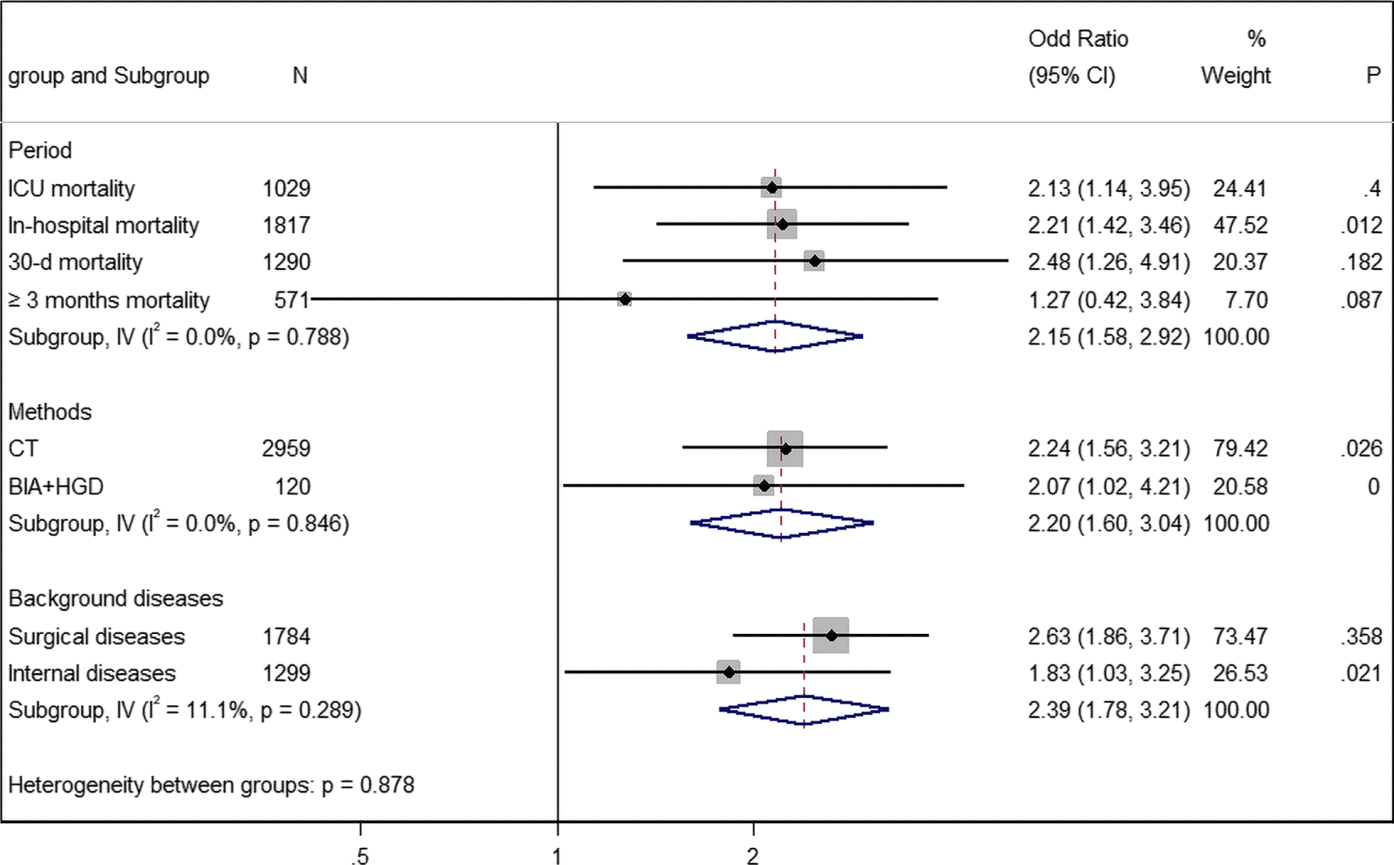
Subgroup analysis of mortality by different periods, methods, and background diseases

Sarcopenia was associated with higher ICU mortality, 30-day mortality, and in-hospital mortality in our study. Among them, five studies involving 1029 patients reported ICU mortality (OR 2.13; 95% CI 1.14, 3.95; *I*^2^ = 60.0%; Fig. 5), seven studies involving 1817 patients reported in-hospital mortality (OR 2.21; 95% CI 1.42, 3.46; *I*^2^ = 63.2%; Fig. 5), and five studies involving 1290 patients reported 30-day mortality (OR 2.48; 95% CI 1.26, 4.91; *I*^2^ = 35.9%; Fig. 5). However, there was no significant difference in ≥ 3-month mortality (OR 1.27; 95% CI 0.42, 3.84; *I*^2^ = 65.8%; Fig. 5) between the two groups. Mortality was also associated with background diseases, and patients with surgical diseases (OR 2.63; 95% CI 1.86, 3.71; *I*^2^ = 9.3%; 9 studies, 1784 cases; Fig. 5) had higher mortality than patients with medical diseases (OR 1.83; 95% CI 1.03, 3.25; *I*^2^ = 69.1%; 4 studies, 1299 cases; Fig. 5). For different diagnosis methods, we found that there was little difference in the effect of sarcopenia diagnosed by CT (OR 2.24; 95% CI 1.56, 3.21; *I*^2^ = 49.6%; 12 studies, 2959 cases; Fig. 5) and BIA plus HGD (OR 2.07; 95% CI 1.02, 4.21; *I*^2^ = 0%; 1 study, 120 cases; Fig. 5) on mortality.

### Meta-regression of mortality

Pooled average age data indicated that it had no impact on all-cause mortality in a meta-regression [regression coefficient 0.005 (95% CI − 0.191 to 0.225), *P* = 0.858, 13 studies, 3079 cases] (Additional file 1: Fig. S3).

### Impact of sarcopenia on the duration of MV, ICU LOS, and hospital LOS

Nine studies involving 1963 participants reported the duration of MV, eleven studies involving 2291 patients reported the ICU LOS, and twelve studies involving 2707 participants reported the hospital LOS. Patients with preexisting sarcopenia had a longer duration of MV (MD = 1.22; 95% CI 0.39, 2.05; *I*^2^ = 97.0%; Additional file 1: Fig. S4), longer days of ICU stay (MD = 1.31; 95% CI 0.43, 2.19; *I*^2^ = 97.0%; Additional file 1: Fig. S5), and hospital stay (MD 2.73; 95% CI 0.58, 4.88; *I*^2^ = 98.0%; Additional file 1: Fig. S6).

### Subgroup analysis of the duration of MV and hospital LOS

Subgroup analyses revealed that sarcopenia, assessed by CT (MD = 1.19; 95% CI 0.99, 1.40; *I*^2^ = 96.0%; Additional file 1: Fig. S7), had a longer duration of MV than sarcopenia, assessed by BIA and HGD (MD = 0.12; 95% CI 0.05, 0.19; *I*^2^ = 95.0%; Additional file 1: Fig. S7). However, for the hospital LOS, subgroup analysis showed the opposite results: sarcopenia assessed by BIA plus HGD (MD = 5.69; 95% CI 4.73, 6.64; *I*^2^ = 99.0%; Additional file 1: Fig. S8) had a longer hospital LOS than that assessed by CT (MD = 1.35; 95% CI 1.08, 1.61; *I*^2^ = 98.0%; Additional file 1: Fig. S8).

### Sensitivity and publication bias analysis

Sensitivity analyses revealed that no individual studies significantly impacted pooled sarcopenia prevalence or risk of mortality (Additional file 1: Fig. S9, Additional file 1: Fig. S10). No publication bias was detected among studies with respect to sarcopenia prevalence (*P* = 0.773, *P* = 0.131, respectively) (Additional file 1: Fig. S11) or risk of mortality (*P* = 0.266, *P* = 0.222, respectively) (Additional file 1: Fig. S12) in patients with MV.

## Discussion

### Primary findings

This study determined that the pooled prevalence of sarcopenia in patients with MV was 43.0% (95% CI 34.0–51.0%; *I*^2^ = 96.7%), which was much higher than that in community-dwelling older adults [42]. Moreover, the pooled analysis indicated that sarcopenia in MV patients is explicitly related to higher short-term mortality risk. We also found that mortality was higher in surgical patients than in medical patients. Additionally, the duration of MV, the days of ICU stay, and the hospital stay were prolonged in these patients.

### Mechanism basis

Whether acute injury or chronic disease, owing to exposure to short-term or long-term oxidative stress and metabolic dysregulation, the prevalence of sarcopenia is higher in MV patients. Patients with sarcopenia often have the following characteristics: (1) Chronic inflammatory condition: Inflammatory factors can directly contribute to decreases in muscle strength and muscle mass among older adults. In chronic disease or acute surgery, first organ functions deteriorate, followed by oxidative stress induction, and some inflammatory cytokines, e.g., tumor necrosis factor-α (TNF-α), C-reactive protein (CRP), interleukin (IL)-8, and IL-6, are often significantly increased [43], contributing to increasing muscle protein degradation and reducing synthesis thereof, leading to net muscle atrophy. (2) Altered hormone levels: Hormonal imbalances characterized by decreased levels of insulin, growth hormone, sex hormones (particularly testosterone), and insulin-like growth factor-1 (IGF-1) [44], together with increases in the levels of angiotensin II, glucocorticoids, and parathyroid hormone [45], and corresponding receptor interactions can ultimately enhance protein degradation and suppress protein synthesis, contributing to sarcopenia onset. (3) Low physical activity: Some patients with chronic disease or acute surgery may need long-term bed rest and lack appetite. This may reduce active and passive exercise, stimulate protein degradation, and reduce protein synthesis, leading to loss of muscle mass [46]. Due to the unfavorable factors mentioned above, patients with sarcopenia who undergo MV often have a poor prognosis.

### Different muscle mass measurement methods

Two predominant strategies were used for sarcopenia classification in patients with MV: definitions based upon independent LMM assessed by CT and LMM plus LMS assessed by BIA and HGD. Sarcopenia prevalence trended upwards in studies that utilized LMM alone (44.0%) compared to studies that used LMM plus LMS (30.0%). This was expected given that the number of diagnosed elements would inevitably decrease the overall rate of sarcopenia detection. According to the latest European Working Group on Sarcopenia in Older People (EWGSOP) update [47], the definition of sarcopenia should consist of evaluating muscle mass and strength and assessing physical performance to gauge severity. However, hand strength and physical performance cannot be evaluated reliably in critical care settings because most MV patients are sedated.

Different diagnostic methods also have a particular impact on prognosis. Our review demonstrated that patients with sarcopenia diagnosed by CT had a longer duration of MV than those diagnosed by BIA and HGD. In contrast, sarcopenia diagnosed by BIA and HGD had a longer hospital LOS than that diagnosed by CT. However, there was little difference in the effect of sarcopenia diagnosed by CT and BIA plus HGD on mortality. The possible reason for the inconsistent results is that there are only two studies on the diagnosis of sarcopenia by BIA and HGD. The inaccurate measurement of grip strength in critically ill patients with MV leads to the biased diagnosis of sarcopenia, which ultimately affects the prognosis of patients.

### Reasons for the heterogeneity

Although subgroup, meta-regression, and sensitivity analyses were performed, heterogeneity was still considerable in the meta-analysis. This is due to the defects of the original literature, which the current systematic review cannot correct. There may be some reasons for the heterogeneity. First, different muscle mass measurement methods are mentioned above. Second, most included studies (15 out of 17) assessed sarcopenia by CT scans, but they were performed at different lumbar vertebrae. Third, our subjects are critically ill patients who may have a variety of primary diseases or acute multiple organ dysfunction. Many confounding factors, such as nutritional status, frailty, physical activity, and sex hormones, may have affected the association between sarcopenia and MV. Finally, differences in race, region, and quality control of the research process between studies may lead to more significant heterogeneity.

### Clinical impacts and gap in knowledge

Our systematic review pooled sarcopenia prevalence among MV patients, offering an up-to-date estimate of sarcopenia prevalence in this patient population. It can guide sample size calculations for future studies related to this topic. Furthermore, in contrast to present acute illness, sarcopenia is understudied in ICU settings. However, baseline LMM has been demonstrated to be a risk factor for poor prognosis in other diseases, including esophageal cancer [48], chronic obstructive pulmonary disease [49], and sepsis in critically ill patients [50]. Our review adds to the growing body of evidence suggesting that LMM is a strong predictor of poor outcomes among patients with MV.

Interestingly, previous studies showed that rehabilitation [51], nutritional support [52], and growth hormone supplementation [53] could improve the prognosis of patients on MV. All of these treatments may improve the patient's muscle mass. This also highlights certain challenges associated with patient clinical management. Should interventions for sarcopenia people with MV be directed toward mitigating key sarcopenic features such as muscle mass? To our knowledge, despite previous studies on nutrition and rehabilitation to improve clinical outcomes, the precise intervention targeting sarcopenia has been the focus of insufficient attention to date in patients with MV. Sarcopenia, however, is generally considered treatable in the context of adult respiratory medicine [54]. More clinical studies are essential to further validate this hypothesis, including novel treatments for sarcopenia, such as nutritional creatine, vitamin D, and β-hydroxy-β-methyl butyrate.

### Strengths and weaknesses

This study has several strengths. First, compared with previous studies [22, 23] on sarcopenia and mortality in critically ill patients, our study's definition of MV was clear. Second, we conducted a comprehensive retrieval to ensure that all relevant original studies were included in our systematic review. Third, the indicators that we analyzed, such as the prevalence of sarcopenia, all-cause mortality, duration of MV, ICU LOS, and hospital LOS, were more comprehensive than those in previous studies.

This review is subject to certain limitations. First, physical function and muscle strength measurements were not available in ICU patients because they were sedated. However, LMM alone for diagnosing sarcopenia is commonly used for patients in the ICU, as published in the surgical literature [55]. Second, most studies were retrospective studies, and only a limited number of studies were included. The researchers could only extract muscle mass data from patients who had CT scans, which may lead to selection bias. Third, the majority of the studies did not have data about health-related quality of life, such as activities of daily living (ADL), instrumental activities of daily living (IADL), and other indicators. Furthermore, the included studies did not provide data on the relationship between comorbidity, frailty, and prognosis. Therefore, we could not perform further analysis.

## Conclusions

### Implications for clinical practice

Sarcopenia is a key clinical condition that affects a large subset of MV patients. Given that sarcopenia adversely impacts mortality and in-hospital adverse outcomes, efforts to identify sarcopenia at early time points when performing clinical assessments of individuals undergoing MV may be warranted to improve patient management and thereby mitigate its impact on MV patients' poor prognoses. However, we acknowledge that the heterogeneity is significant in our study. Therefore, the results should be interpreted cautiously.

### Implications for research

There is a need to conduct a prospective cohort study with a unified and standard diagnostic method and a large sample size for sarcopenia. Long-term qualitative outcomes should be considered in future studies.

## Supplementary Information

**Additional file 1: Table S1.** PRISMA 2020 Checklist. **Table S2.** Search strategy by MEDLINE, Embase, The Cochrane Database of Systematic Reviews, and The Cochrane Central Register of Controlled Trials via Ovid SP. **Table S3.** The impact of sarcopenia on mortality in patients with MV. **Table S4.** The reasons for the exclusion of full-text articles. **Table S5.** The details of diagnosis criteria and cutoff points of each study. **Table S6.** Result of the Newcastle–Ottawa scale quality assessment. **Fig. S1.** Subgroup analysis of sarcopenia prevalence at different CT sites. **Fig. S2.** Meta-regression of the effect of average age on sarcopenia prevalence. **Fig. S3.** Meta-regression of the effect of average age on mortality. **Fig. S4.** The duration of mechanical ventilation. **Fig. S5.** The length of ICU stay. **Fig. S6.** The length of hospital stay. **Fig. S7.** Subgroup analysis of effects with different diagnostic methods on the duration of mechanical ventilation. **Fig. S8.** Subgroup analysis of effects with different diagnostic methods on the length of hospital stay. **Fig. S9.** The sensitivity analysis of prevalence. **Fig. S10.** The sensitivity analysis for ORs between sarcopenia and mortality. **Fig. S11.** Begg's and Egger's tests for publication bias of prevalence. **Fig. S12.** Begg's and Egger's tests for publication bias of mortality.

## Data Availability

The datasets used and/or analyzed during the current study are available from the corresponding author on reasonable request.

## Abbreviations

ADL: Activities of daily living
BIA: Bioelectrical impedance analysis
BIVA: Bioelectrical impedance vector analysis
BMI: Body mass index
CAP: Community-acquired pneumonia
CCI: Charlson Comorbidity Index
CI: Confidence intervals
COPD: Chronic obstructive pulmonary disease
COVID-19: Coronavirus disease 2019
CRP: C-reactive protein
CT: Computed tomography
DTW: Difficult-to-wean
DXA: Dual-energy X-ray absorptiometry
EWGSOP: European Working Group on Sarcopenia in Older People
GS: Gait speed
HGD: Handgrip dynamometry
HGS: Hand-grip strength
ICU: Intensive care unit
ICU LOS: The length of intensive care unit stay
IGF-1: Insulin-like growth factor-1
IL: Interleukin
LMM: Low muscle mass
LMS: Low muscle strength
LOS: The length of hospital stay
LPF: Low physical function
LPM: Lumbar paraspinal muscle
MV: Mechanical ventilation
NOS: Newcastle–Ottawa Scale
NR: Not reported
OR: Odds ratio
PLVI: Psoas lumbar vertebral index
PMCSA: Cross-sectional area of the pectoralis muscle at the fourth vertebral region
SMA: Skeletal muscle area
SOFA: Sequential Organ Failure Assessment
TNF-α: Tumor necrosis factor-α
TPA: Total psoas muscle area
TPM: Thoracic paravertebral muscle
