# Supplementary material for: Prevalence and prognostic value of preexisting sarcopenia in patients with mechanical ventilation: a systematic review and meta-analysis
Source: Crit Care. 2022 May 16;26:140. doi: 10.1186/s13054-022-04015-y (PMC9109453; doi:10.1186/s13054-022-04015-y)
Supplement: Supplementary file 1 — Additional file 1: Table S1. PRISMA 2020 Checklist. Table S2. Search strategy by MEDLINE, Embase, The Cochrane Database of Systematic Reviews, and The Cochrane Central Register of Controlled Trials via Ovid SP. Table S3. The impact of sarcopenia on mortality in patients with MV. Table S4. The reasons for the exclusion of full-text articles. Table S5. The details of diagnosis criteria and cutoff points of each study. Table S6. Result of the Newcastle–Ottawa scale quality assessment. Fig. S1. Subgroup analysis of sarcopenia prevalence at different CT sites. Fig. S2. Meta-regression of the effect of average age on sarcopenia prevalence. Fig. S3. Meta-regression of the effect of average age on mortality. Fig. S4. The duration of mechanical ventilation. Fig. S5. The length of ICU stay. Fig. S6. The length of hospital stay. Fig. S7. Subgroup analysis of effects with different diagnostic methods on the duration of mechanical ventilation. Fig. S8. Subgroup analysis of effects with different diagnostic methods on the length of hospital stay. Fig. S9. The sensitivity analysis of prevalence. Fig. S10. The sensitivity analysis for ORs between sarcopenia and mortality. Fig. S11. Begg's and Egger's tests for publication bias of prevalence. Fig. S12. Begg's and Egger's tests for publication bias of mortality. [file 13054_2022_4015_MOESM1_ESM.docx]

**Table S1: PRISMA 2020 Checklist**

| **Section and Topic** | **Item #** | **Checklist item** | **Location**  **where item is reported** |
| --- | --- | --- | --- |
| **TITLE** | | |  |
| Title | 1 | Identify the report as a systematic review. | Pg. 1, Lines 2 |
| **ABSTRACT** | | |  |
| Abstract | 2 | See the PRISMA 2020 for Abstracts checklist. | Pg. 2 |
| **INTRODUCTION** | | |  |
| Rationale | 3 | Describe the rationale for the review in the context of existing knowledge. | Pg. 4-5, Lines 73-115 |
| Objectives | 4 | Provide an explicit statement of the objective(s) or question(s) the review addresses. | Pg. 5, Lines 107-115 |
| **METHODS** | | |  |
| Eligibility criteria | 5 | Specify the inclusion and exclusion criteria for the review and how studies were grouped for the syntheses. | Pg. 6, Lines 128-138 |
| Information sources | 6 | Specify all databases, registers, websites, organizations, reference lists and other sources searched or consulted to identify studies. Specify the date when each source was last searched or consulted. | Pg. 5-6, Lines 117-127 |
| Search strategy | 7 | Present the full search strategies for all databases, registers and websites, including any filters and limits used. | Table S2 |
| Selection process | 8 | Specify the methods used to decide whether a study met the inclusion criteria of the review, including how many reviewers screened each record and each report retrieved, whether they worked independently, and if applicable, details of automation tools used in the process. | Pg. 6-7, Lines 139-146 |
| Data collection process | 9 | Specify the methods used to collect data from reports, including how many reviewers collected data from each report, whether they worked independently, any processes for obtaining or confirming data from study investigators, and if applicable, details of automation tools used in the process. | Pg. 6-7, Lines 146-149 |
| Data items | 10a | List and define all outcomes for which data were sought. Specify whether all results that were compatible with each outcome domain in each study were sought (e.g. for all measures, time points, analyses), and if not, the methods used to decide which results to collect. | Pg. 7, Lines 156-169 |
|  | 10b | List and define all other variables for which data were sought (e.g. participant and intervention characteristics, funding sources). Describe any assumptions made about any missing or unclear information. | Table1 |
| Study risk of bias assessment | 11 | Specify the methods used to assess risk of bias in the included studies, including details of the tool(s) used, how many reviewers assessed each study and whether they worked independently, and if applicable, details of automation tools used in the process. | Pg. 9, Lines 201-205 |
| Effect measures | 12 | Specify for each outcome the effect measure(s) (e.g. risk ratio, mean difference) used in the synthesis or presentation of results. | Pg. 8, Lines 178-185 |
| Synthesis methods | 13a | Describe the processes used to decide which studies were eligible for each synthesis (e.g. tabulating the study intervention characteristics and comparing against the planned groups for each synthesis (item #5)). | Table 1 and Figure 1 |
|  | 13b | Describe any methods required to prepare the data for presentation or synthesis, such as handling of missing summary statistics, or data conversions. | Pg. 8, Lines 170-177 |
|  | 13c | Describe any methods used to tabulate or visually display results of individual studies and syntheses. | Pg. 8, Lines 170-177 |
|  | 13d | Describe any methods used to synthesize results and provide a rationale for the choice(s). If meta-analysis was performed, describe the model(s), method(s) to identify the presence and extent of statistical heterogeneity, and software package(s) used. | Pg. 8, Lines 171-185 |
|  | 13e | Describe any methods used to explore possible causes of heterogeneity among study results (e.g. subgroup analysis, meta-regression). | Pg. 8-9, Lines 186-200 |
|  | 13f | Describe any sensitivity analyses conducted to assess robustness of the synthesized results. | Pg. 9, Lines 201-203; |
| Reporting bias assessment | 14 | Describe any methods used to assess risk of bias due to missing results in a synthesis (arising from reporting biases). | Pg. 9, Lines 203-205 |

| **Section and Topic** | **Item #** | **Checklist item** | **Location where item**  **is reported** |
| --- | --- | --- | --- |
| Certainty assessment | 15 | Describe any methods used to assess certainty (or confidence) in the body of evidence for an outcome. | None |
| **RESULTS** | | |  |
| Study selection | 16a | Describe the results of the search and selection process, from the number of records identified in the search to the number of studies included in the review, ideally using a flow diagram. | Figure 1 |
|  | 16b | Cite studies that might appear to meet the inclusion criteria, but which were excluded, and explain why they were excluded. | Table S4 |
| Study characteristics | 17 | Cite each included study and present its characteristics. | Table 1 |
| Risk of bias in studies | 18 | Present assessments of risk of bias for each included study. | Table S6 |
| Results of individual studies | 19 | For all outcomes, present, for each study: (a) summary statistics for each group (where appropriate) and (b) an effect estimate and its precision (e.g. confidence/credible interval), ideally using structured tables or plots. | Figure 2-4; Figure S1-S8 |
| Results of syntheses | 20a | For each synthesis, briefly summarise the characteristics and risk of bias among contributing studies. | Pg. 9-10, Lines 216-139 |
|  | 20b | Present results of all statistical syntheses conducted. If meta-analysis was done, present for each the summary estimate and its precision (e.g. confidence/credible interval) and measures of statistical heterogeneity. If comparing groups, describe the direction of the effect. | Pg. 10-13; Lines 240-309 |
|  | 20c | Present results of all investigations of possible causes of heterogeneity among study results. | Pg. 11, Lines 244-261; Pg. 12, Lines 266-286; Pg. 13, Lines 295-306 |
|  | 20d | Present results of all sensitivity analyses conducted to assess the robustness of the synthesized results. | Pg. 13, Lines 304-306 |
| Reporting biases | 21 | Present assessments of risk of bias due to missing results (arising from reporting biases) for each synthesis assessed. | Pg. 13, Lines 306-309 |
| Certainty of evidence | 22 | Present assessments of certainty (or confidence) in the body of evidence for each outcome assessed. | None |
| **DISCUSSION** | | |  |
| Discussion | 23a | Provide a general interpretation of the results in the context of other evidence. | Pg. 13-18 |
|  | 23b | Discuss any limitations of the evidence included in the review. | Pg. 14, Lines 312-318 |
|  | 23c | Discuss any limitations of the review processes used. | Pg. 18, Lines 413-425 |
|  | 23d | Discuss implications of the results for practice, policy, and future research. | Pg. 18-19, Lines 427-439 |
| **OTHER INFORMATION** | | |  |
| Registration and protocol | 24a | Provide registration information for the review, including register name and registration number, or state that the review was not registered. | Pg. 5, Lines 120-121 |
|  | 24b | Indicate where the review protocol can be accessed, or state that a protocol was not prepared. | Pg. 5, Lines 120-121 |
|  | 24c | Describe and explain any amendments to information provided at registration or in the protocol. | None |
| Support | 25 | Describe sources of financial or non-financial support for the review, and the role of the funders or sponsors in the review. | Pg. 20, Lines 470-478 |
| Competing interests | 26 | Declare any competing interests of review authors. | Pg. 21, Lines 493-494 |
| Availability of data, code and other materials | 27 | Report which of the following are publicly available and where they can be found: template data collection forms; data extracted from included studies; data used for all analyses; analytic code; any other materials used in the review. | Table 1 and Table S3 |

*From:* Page MJ, McKenzie JE, Bossuyt PM, et al. The PRISMA 2020 statement: an updated guideline for reporting systematic reviews. *BMJ* 2021;372:n71. doi:10.1136/bmj.n71

For more information, visit: **www.prisma-statement.org**.

Table S2: Search strategy by MEDLINE, EMBASE, The Cochrane Database of Systematic Reviews, and The Cochrane Central Register of Controlled Trials via Ovid SP

| - - 1. exp sarcopenia/ |
| --- |
| - - 1. (sarcopeni* or myopeni* or dynaponi*).tw. |
| - - 1. ((muscle or muscular) adj2 (atroph* or wasting* or weak* or loss*)).tw. |
| - - 1. or/1-3 |
| - - 1. exp ventilation/ |
| - - 1. (respira* or ventila*).tw. |
| - - 1. ((artificial or assisted or mechanical) adj2 (ventila* or respira*)).tw. |
| - - 1. or/5-7 |
| - - 1. exp animals/ not humans.sh. |
| - - 1. (4 and 8) not 9 |
| - - 1. remove duplicates from 10 |

Table S3: The impact of sarcopenia on mortality in patients with MV.

| First author and year | OR (95% CI) without adjustment | OR (95% CI) with adjustment | Adjustment factors | Different time of mortality |
| --- | --- | --- | --- | --- |
| Kou, H. W. 2019 | 5.071 (1.06–24.28) |  |  | ICU mortality |
| Moisey, L. L.2013 | 2.91 (1.12-7.56) |  |  | In-hospital mortality |
| Weijs,P.J. 2014 | 2.72 (1.24-5.97)  4.32 (2.21-8.8)  4.6 (1.73-2.28) |  |  | ICU mortality  In-hospital mortality  30-day mortality |
| Moctezuma-Velázquez, P. 2021 | 0.95 (0.54-1.65) |  |  | In-hospital mortality |
| Han, J. W. 2021 | 3.957 (1.886-8.302) |  |  | In-hospital mortality |
| Ebbeling, L. 2013 | 1.2 (0.44-3.6) |  |  | In-hospital mortality |
| Joyce 2020 | 0.57 (0.22-1.5)  1.57 (0.71-3.47) |  |  | ICU-mortality  30-day mortality |
| Akahoshi, T. 2016 | 1.19 (0.10-13.73) |  |  | 30-day mortality |
| Ng, Cc 2020 | 3.17 (1.56-6.41)  2.415 (1.16–5.03) |  |  | ICU-mortality  In-hospital mortality |
| Xi, F. 2021 | 0.6 (0.07-5.21)  0.66 (0.22-1.99) |  |  | 30-day mortality  90-day mortality |
| Vongchaiudomchoke, W. 2021 | 2.06 (1.02-4.17) | 2.07 (1.02-4.22) | SOFA scores, serum albumin, nosocomial infection, duration of mechanical ventilation, and hospital length of stay | 120-day mortality |
| Moon, S. W. 2021 | 2.17 (1.07-4.39)  1.68 (1.04–2.74) | In-hospital mortality 2.06 (1.23–3.47) | age, sex, BMI, CCI, whether the patient  underwent intubation, and SOFA score | ICU-mortality  In-hospital mortality |
| Yun Ji. 2018 | 6.8 (2.6–17.8) | 4.6 (1.7–12.8) | age, use of vasopressor, mixed organism, and Acute Physiology and Chronic Health Evaluation II score | 30-day mortality |

BMI: Body mass index; CCI: Charlson comorbidity index; CI: Confidence intervals; ICU: Intensive care unit; MV: Mechanical ventilation; OR: Odds ratio; SOFA: Sequential organ failure assessment.

Table S4: The reasons for the exclusion of full-text articles

| Study | Reason for the exclusion |
| --- | --- |
| Baldwin 2014 [1] | Not reported the date of prevalence or prognosis |
| Borges 2019 [2] | Not reported the date of prevalence or prognosis |
| Shi 2021 [3] | Not reported the date of prevalence or prognosis |
| Turton 2016 [4] | Not reported the date of prevalence or prognosis |
| Doorduin 2018 [5] | Not reported the date of prevalence or prognosis |
| Brunello 2010 [6] | Not reported the date of prevalence or prognosis |
| Lee, Z. Y. 2021 [7] | Not reported the diagnosis criteria of sarcopenia |
| Sheean 2010 [8] | Not reported the date of prevalence or prognosis |
| Loss 2015 [9] | Not reported the date of prevalence or prognosis |
| Chang 2005 [10] | Not reported the date of prevalence or prognosis |
| Silva 2018 [11] | Not reported the date of prevalence or prognosis |
| Twose 2018 [12] | Not reported the date of prevalence or prognosis |
| Kayim Yildiz 2021 [13] | Exclusion study types: A case report |
| Schepens 2015 [14] | Not reported the diagnostic criteria of sarcopenia |
| Carambula 2021 [15] | Exclusion study types: editorial |
| Uchiyama 2021 [16] | Exclusion study types: case report |
| Baggerman, M. R. 2020 [17] | Not reported whether or not with mechanical ventilation |
| Shibahashi, K. 2017 [18] | Not reported whether or not with mechanical ventilation |
| Toledo, D. O. 2018 [19] | Not reported whether or not with mechanical ventilation |
| Cho, W. H. 2020 [20] | Not reported whether or not with mechanical ventilation |
| Hwang, F. 2019 [21] | Not reported whether or not with mechanical ventilation |
| Tanabe, C. 2019 [22] | Not reported whether or not with mechanical ventilation |
| Raurell-Torreda, M. 2021 [23] | Patients with ICU-acquired weakness |
| De Jonghe, B. 2002 [24] | Patients with ICU-acquired weakness |
| Medrinal, C. 2021 [25] | Patients with ICU-acquired weakness |

Table S5: The details of diagnosis criteria and cut-off points of each study

| Measurement Methods | Cut-off Values | Studies |
| --- | --- | --- |
| CT | L3 SMI ≤ 38.5 cm^2^ /m^2^ for women and ≤ 52.4 cm^2^ /m^2^ for men | Sheean 2014 |
|  | L3 SMI less than 38.9 cm^2^ /m^2^ for females and less than 55.4 cm^2^ /m^2^ for males | Moisey 2013 |
|  | L3 SMI < 55 cm^2^ /m^2^ in males and < 39 cm^2^ /m^2^ in females | Doolittle 2021 |
|  | L3 SMI ≤ 49 cm^2^ /m^2^ for men and of 31 cm^2^ /m^2^ for women | Woo 2020 |
|  | L3 ≤ 42.08 cm^2^ /m^2^ for men and 37.35 cm^2^ /m^2^ for women | Xi, F. 2021 |
|  | T12 SMI less than 42.6 cm^2^ /m^2^ and less than 30.6 cm^2^ /m^2^ in men and women | Moctezuma-Velázquez, P. 2021 |
|  | L3 SMA < 110 cm^2^ for females and 170 cm^2^ for males | Weijs 2014;  Joyce 2020 |
|  | L3 SMA < 80 % estimated SMA | Akahoshi, T. 2016 |
|  | TPA less than 385 mm^2^ /m^2^ for female or 545 mm^2^ /m^2^ for male | Kou 2019 |
|  | L1 SMI ≤ 45 cm^2^ /m^2^ in males and 40 cm^2^ /m^2^ in females | Han, J. W. 2021 |
|  | L3 SMI ≤ 40.8 cm^2^ /m^2^ for males and 34.9 cm^2^ /m^2^ for females | Yun Ji. 2018 |
|  | PMCSA lower than 26.5 cm^2^ in men, and 18.3 cm^2^ in women, respectively. | Moon, S. W. 2021 |
|  | Psoas: L4 Vertebral Index < 50 percentile of PLVI (≤ 0.83) | Ebbeling, L. 2013 |
|  | L3 < 42.0 cm^2^/m^2^ for both sexes | Ng, Cc 2020 |
| BIA | LMM < 7.0 kg/m^2^ for men and <5.7 kg/m^2^ for women | Yuenyongchaiwat 2020; Vongchaiudomchoke, W. 2021 |
| HGD | HGS < 26 kg for males and < 18 kg for females | Yuenyongchaiwat 2020; Vongchaiudomchoke, W. 2021 |

BIA: Bioelectrical impedance analysis; CT: Computed tomography; HGD: handgrip dynamometer; HGS: Hand-grip strength; LMM: Low muscle mass; PLVI: Psoas lumbar vertebral index; PMCSA: Cross-sectional area of the pectoralis muscle at the fourth vertebral region; SMI: SMA: skeletal muscle area; Skeletal muscle index; TPA: Total psoas muscle area.

Table S6: Result of the Newcastle-Ottawa scale quality assessment.

| Newcastle-Ottawa scale | Selection (1) |  |  |  | Comparability (2) | Outcome (3) |  |  | Total |
| --- | --- | --- | --- | --- | --- | --- | --- | --- | --- |
|  | Representativeness  of the exposed cohort | Selection of the non-exposed cohort | Ascertainment of exposure | Outcomes were not present at study initiation | Comparability of cohorts on the basis of the design or analysis | Assessment of outcome | Was follow-up long enough for outcome to occur | Adequacy  of follow-up |  |
| Moisey 2013 | 1 | 1 | 1 | 1 | 2 | 1 | 0 | 1 | 8 |
| Kou 2019 | 1 | 1 | 1 | 1 | 1 | 1 | 0 | 1 | 7 |
| Doolittle 2021 | 1 | 1 | 1 | 0 | 1 | 1 | 0 | 1 | 6 |
| Yuenyongchaiwat 2020 | 1 | 1 | 1 | 0 | 1 | 1 | 0 | 1 | 6 |
| Woo 2020 | 1 | 1 | 1 | 1 | 1 | 1 | 0 | 1 | 7 |
| Sheean 2014 | 0 | 0 | 0 | 1 | 0 | 1 | 0 | 0 | 2 |
| Weijs 2014 | 1 | 1 | 1 | 1 | 1 | 1 | 0 | 1 | 7 |
| Yun Ji. 2018 | 1 | 1 | 1 | 1 | 1 | 1 | 1 | 1 | 8 |
| Ebbeling, L. 2013 | 1 | 1 | 1 | 1 | 1 | 1 | 1 | 0 | 7 |
| Joyce 2020 | 1 | 1 | 1 | 1 | 1 | 1 | 0 | 0 | 6 |
| Akahoshi, T. 2016 | 1 | 1 | 1 | 1 | 1 | 1 | 0 | 0 | 6 |
| Ng, Cc 2020 | 1 | 1 | 1 | 1 | 1 | 1 | 1 | 0 | 7 |
| Moctezuma-Velázquez, P. 2021 | 1 | 1 | 1 | 1 | 1 | 1 | 0 | 0 | 6 |
| Moon, S. W. 2021 | 1 | 1 | 1 | 1 | 0 | 1 | 0 | 0 | 5 |
| Han, J. W. 2021 | 1 | 1 | 1 | 1 | 1 | 0 | 0 | 0 | 5 |
| Vongchaiudomchoke, W. 2021 | 1 | 1 | 1 | 1 | 1 | 1 | 1 | 1 | 8 |
| Xi, F. 2021 | 1 | 1 | 1 | 1 | 1 | 1 | 0 | 0 | 6 |


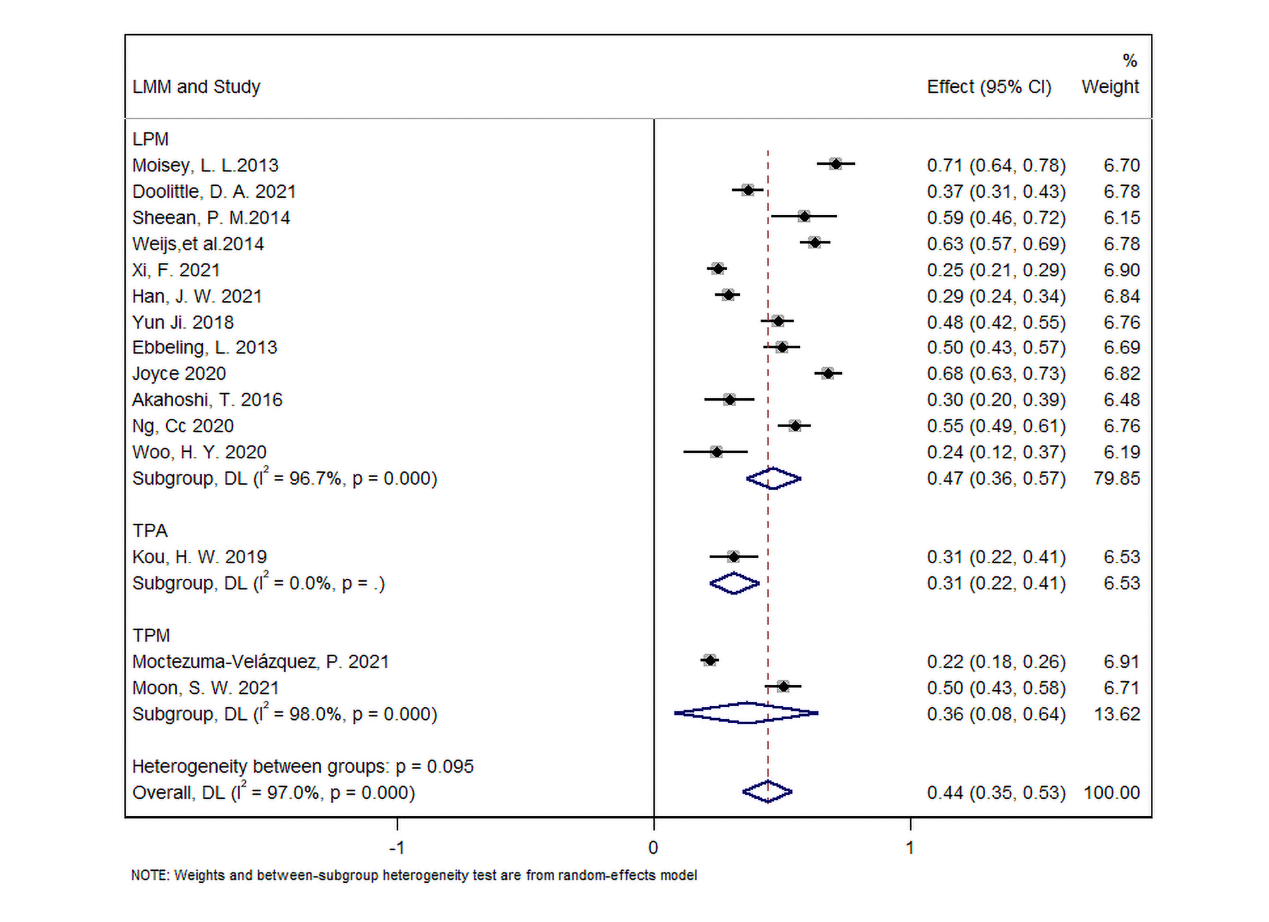


Fig. S1: Subgroup analysis of sarcopenia prevalence with different CT sites.


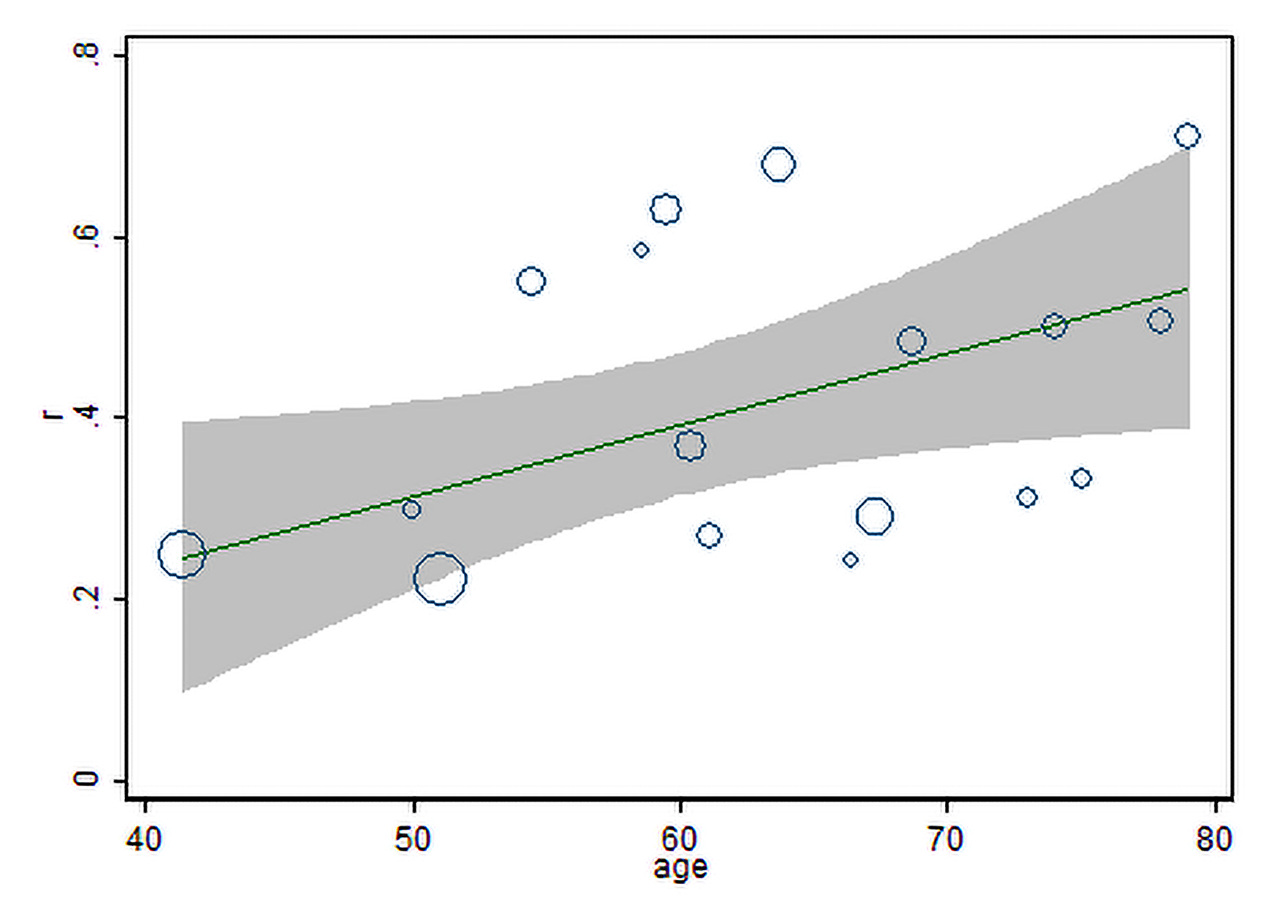


Fig. S2: Meta-regression of the effect of average age on sarcopenia prevalence.

Regression coefficient 0.005 (95% CI: −0.003 to 0.013), P = 0.165. Circle diameters reflective of proportional study sample size.


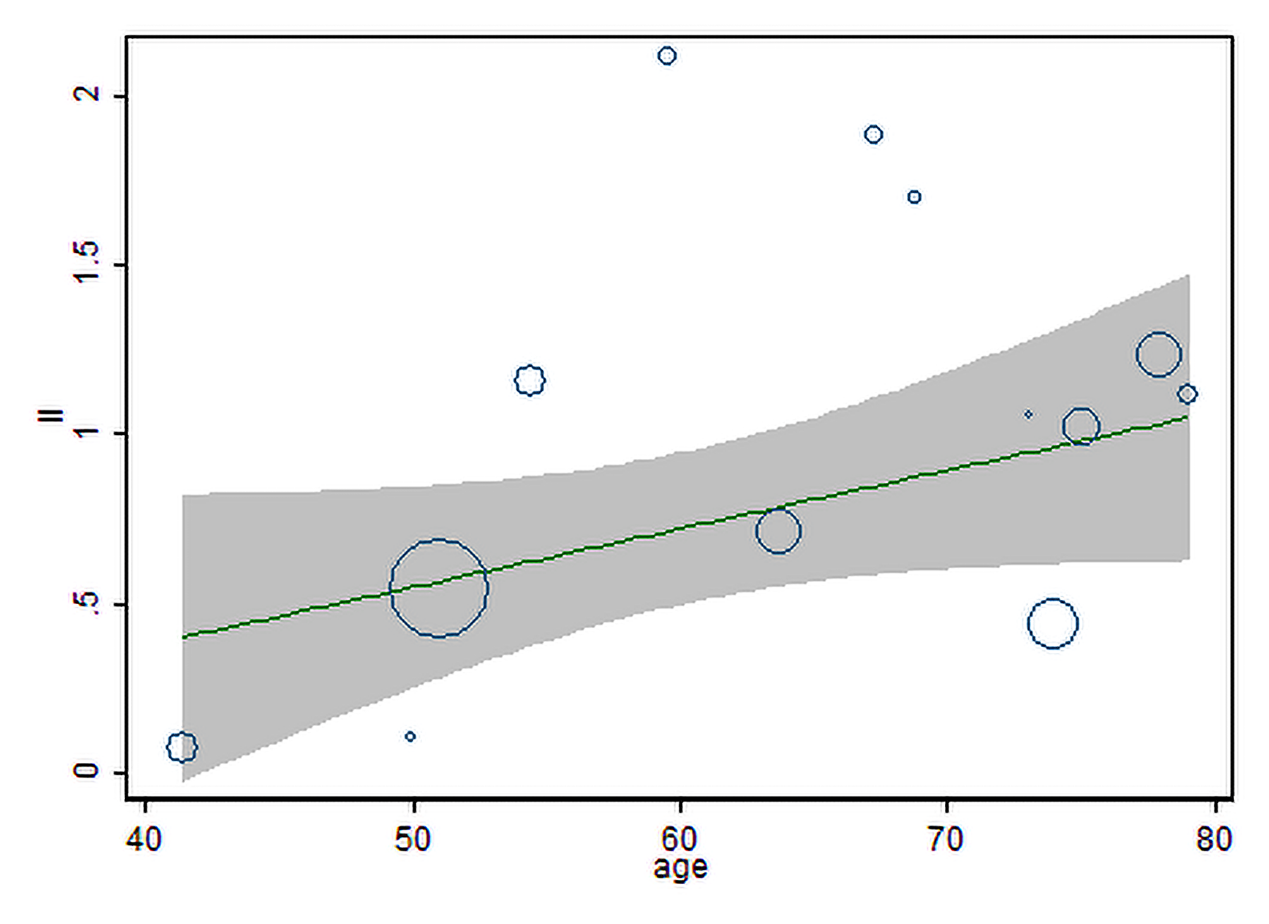


Fig. S3: Meta-regression of the effect of average age on mortality.

Regression coefficient 0.005 (95% CI: −0.191 to 0.225), P = 0.858. Circle diameters reflective of proportional study sample size.





Fig. S4: The duration of mechanical ventilation.





Fig. S5: The length of ICU stay.





Fig. S6: The length of hospital stay.





Fig. S7: Subgroup analysis of effects with different diagnostic methods on the duration of mechanical ventilation.





Fig. S8: Subgroup analysis of effects with different diagnostic methods on the length of hospital stay.


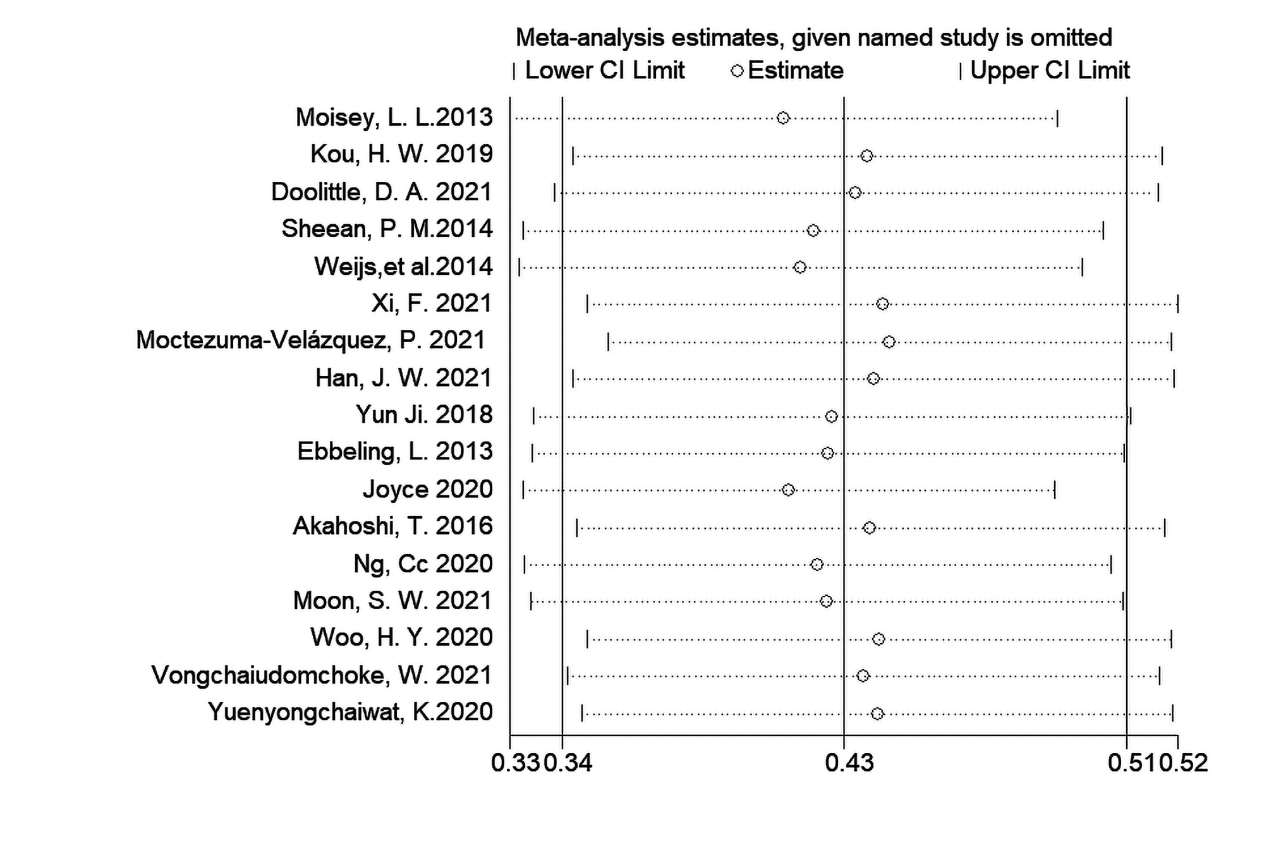
 Fig. S9: The sensitivity analysis of prevalence.


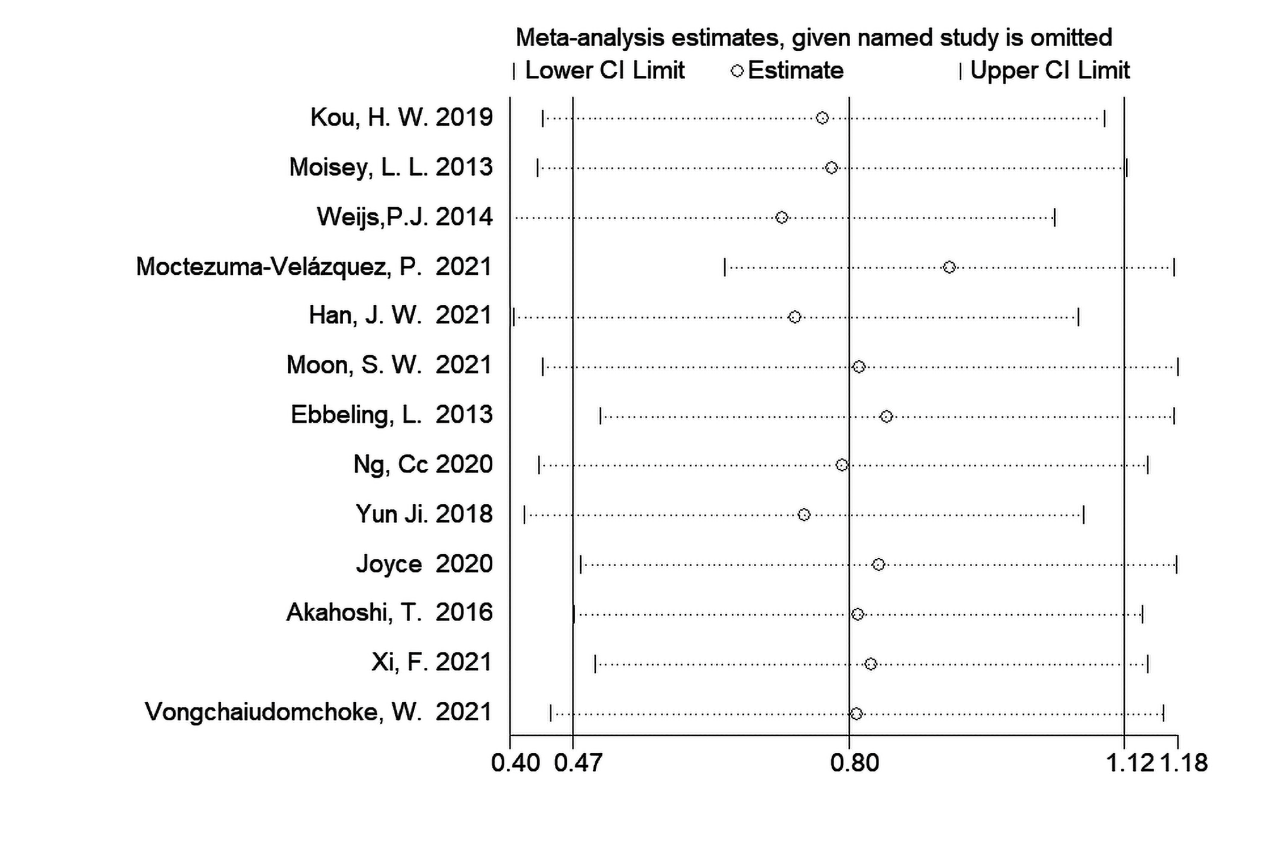


Fig. S10: The sensitivity analysis for ORs between sarcopenia and mortality.


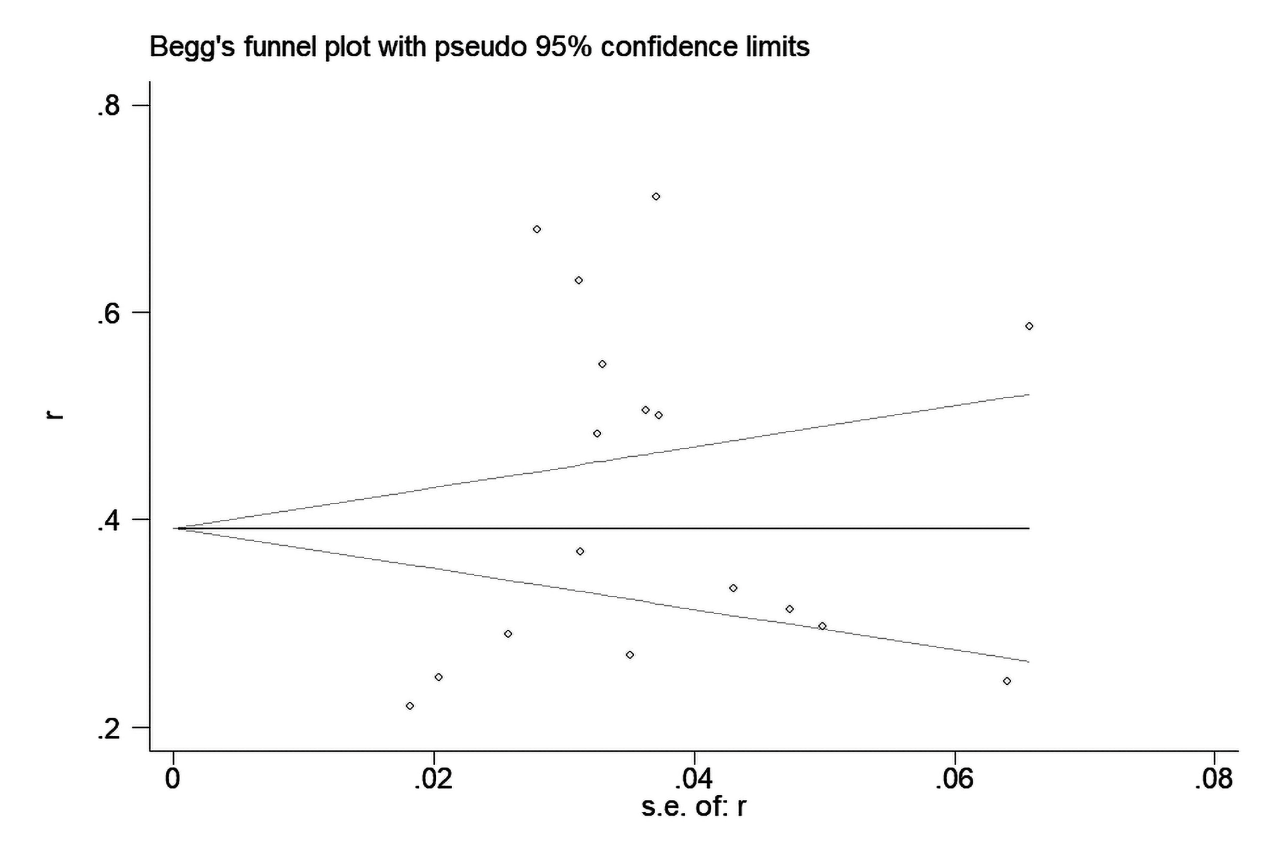


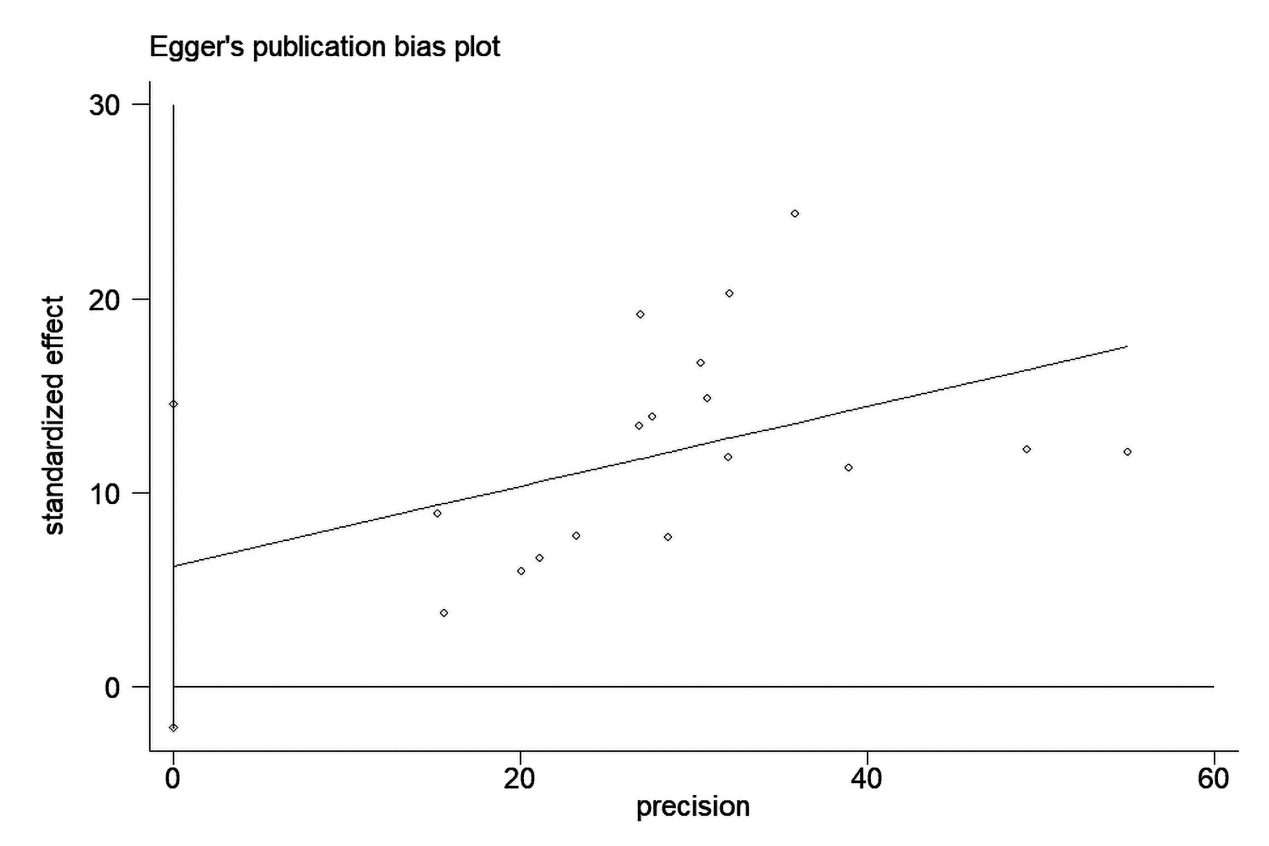


Fig. S11: Begg's and Egger's test for publication bias of prevalence.


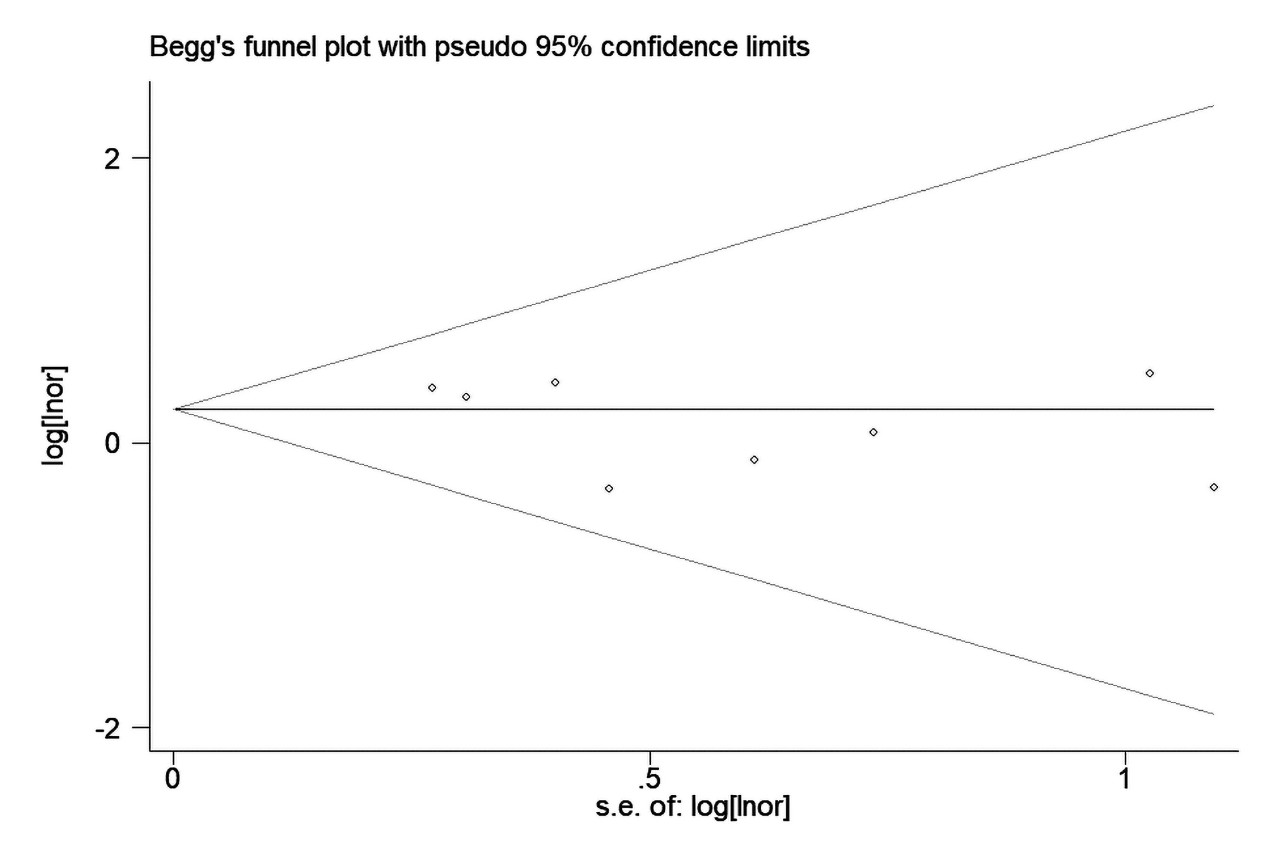


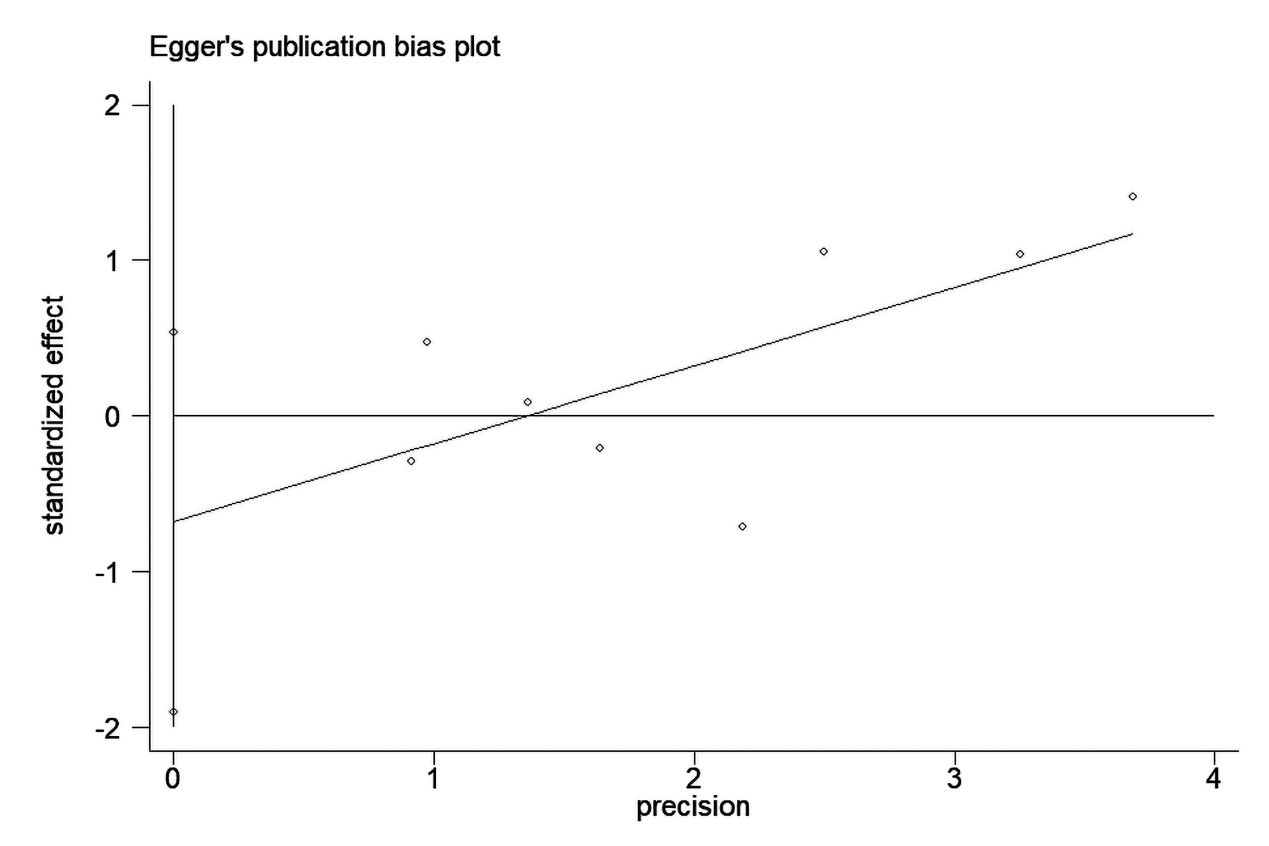


Fig. S12: Begg's and Egger's test for publication bias of mortality.

References:

1. Baldwin, C.E. & Bersten, A.D. Alterations in respiratory and limb muscle strength and size in patients with sepsis who are mechanically ventilated. *Physical Therapy* **94**, 68-82 (2014).

2. Borges, R.C. & Soriano, F.G. Association Between Muscle Wasting and Muscle Strength in Patients Who Developed Severe Sepsis And Septic Shock. *Shock* **51**, 312-320 (2019).

3. Shi, Z.H.*, et al.* Changes in Respiratory Muscle Thickness during Mechanical Ventilation. *Anesthesiology* **12**, 12 (2020).

4. Turton, P., Hay, R., Taylor, J., McPhee, J. & Welters, I. Human limb skeletal muscle wasting and architectural remodeling during five to ten days intubation and ventilation in critical care - an observational study using ultrasound. *BMC Anesthesiology* **16**, 119 (2016).

5. Doorduin, J.*, et al.* Respiratory Muscle Effort during Expiration in Successful and Failed Weaning from Mechanical Ventilation. *Anesthesiology* **129**, 490-501 (2018).

6. Brunello, A.G.*, et al.* Usefulness of a clinical diagnosis of ICU-acquired paresis to predict outcome in patients with SIRS and acute respiratory failure. *Intensive Care Medicine* **36**, 66-74 (2010).

7. Lee, Z.Y.*, et al.* Association between ultrasound quadriceps muscle status with premorbid functional status and 60-day mortality in mechanically ventilated critically ill patient: A single-center prospective observational study. *Clinical Nutrition* **40**, 1338-1347 (2021).

8. Sheean, P.M., Peterson, S.J., Gurka, D.P. & Braunschweig, C.A. Nutrition assessment: the reproducibility of subjective global assessment in patients requiring mechanical ventilation. *European Journal of Clinical Nutrition* **64**, 1358-1364 (2010).

9. Loss, S.H.*, et al.* The reality of patients requiring prolonged mechanical ventilation: a multicenter study. *Revista Brasileira de Terapia Intensiva* **27**, 26-35 (2015).

10. Chang, A.T., Boots, R.J., Brown, M.G., Paratz, J. & Hodges, P.W. Reduced inspiratory muscle endurance following successful weaning from prolonged mechanical ventilation. *Chest* **128**, 553-559 (2005).

11. Silva, P.E.*, et al.* Neuromuscular electrophysiological disorders and muscle atrophy in mechanically-ventilated traumatic brain injury patients: New insights from a prospective observational study. *Journal of Critical Care* **44**, 87-94 (2018).

12. Twose, P., Jones, U. & Wise, M.P. Effect of hypercapnia on respiratory and peripheral skeletal muscle loss during critical illness - A pilot study. *Journal of Critical Care* **45**, 105-109 (2018).

13. Kayim Yildiz, O., Yildiz, B., Avci, O., Hasbek, M. & Kanat, S. Clinical, Neurophysiological and Neuroimaging Findings of Critical Illness Myopathy After COVID-19. *Cureus* **13**, e13807 (2021).

14. Schepens, T.*, et al.* The course of diaphragm atrophy in ventilated patients assessed with ultrasound: a longitudinal cohort study. *Critical Care (London, England)* **19**, 422 (2015).

15. Carambula, A., Pereyra, S., Barbato, M. & Angulo, M. Combined Diaphragm and Limb Muscle Atrophy Is Associated With Increased Mortality in Mechanically Ventilated Patients: A Pilot Study. *Archivos De Bronconeumologia* **57**, 377-379 (2021).

16. Uchiyama, Y.*, et al.* COVID-19 Patient Returned to Work after Long Hospitalization and Follow-up: A Case Report. *Progress in Rehabilitation Medicine* **6**, 20210025 (2021).

17. Baggerman, M.R.*, et al.* Muscle wasting associated co-morbidities, rather than sarcopenia are risk factors for hospital mortality in critical illness. *J Crit Care* **56**, 31-36 (2020).

18. Shibahashi, K., Sugiyama, K., Kashiura, M. & Hamabe, Y. Decreasing skeletal muscle as a risk factor for mortality in elderly patients with sepsis: a retrospective cohort study. *Journal of intensive care*, 5-8 (2017).

19. Toledo, D.O.*, et al.* The use of computed tomography images as a prognostic marker in critically ill cancer patients. *Clin Nutr ESPEN* **25**, 114-120 (2018).

20. Cho, W.H.*, et al.* Prognostic Value of Sarcopenia for Long-Term Mortality in Extracorporeal Membrane Oxygenation for Acute Respiratory Failure. *ASAIO J* **66**, 367-372 (2020).

21. Hwang, F.*, et al.* Sarcopenia is Predictive of Functional Outcomes in Older Trauma Patients. *Cureus* **11(11)**, e6154 (2019).

22. Tanabe, C.*, et al.* Association of Brain Atrophy and Masseter Sarcopenia With 1-Year Mortality in Older Trauma Patients. *JAMA Surg* **154**, 716-723 (2019).

23. Raurell-Torreda, M.*, et al.* Care and treatments related to intensive care unit-acquired muscle weakness: A cohort study. *Australian Critical Care* **01**, 01 (2021).

24. De Jonghe, B.*, et al.* Paresis acquired in the intensive care unit: a prospective multicenter study. *JAMA* **288**, 2859-2867 (2002).

25. Medrinal, C.*, et al.* Muscle weakness, functional capacities and recovery for COVID-19 ICU survivors. *BMC Anesthesiology* **21**, 64 (2021).
